# Alveolar Microdynamics during Tidal Ventilation in Live Animals Imaged by SPring‐8 Synchrotron

**DOI:** 10.1002/advs.202306256

**Published:** 2024-07-03

**Authors:** Min Woo Kim, Seung Hyeon Yu, Un Yang, Ryota Nukiwa, Hyeon Jung Cho, Nam Seop Kwon, Moon Jung Yong, Nam Ho Kim, Sang Hyeon Lee, Jun Ho Lee, Jae Hong Lim, Yoshiki Kohmura, Tatsuya Ishikawa, Frank S. Henry, Yumiko Imai, Seung Soo Oh, Hyung Ju Hwang, Akira Tsuda, Jung Ho Je

**Affiliations:** ^1^ School of Interdisciplinary Bioscience and Bioengineering Pohang University of Science and Technology (POSTECH) Pohang 37673 South Korea; ^2^ Pohang Accelerator Laboratory (PAL) POSTECH Pohang 37673 South Korea; ^3^ Department of Mathematics POSTECH Pohang 37673 South Korea; ^4^ Department of Materials Science and Engineering POSTECH Pohang 37673 South Korea; ^5^ National Institutes of Biomedical Innovation Health and Nutrition Infection Medical Information Laboratory Osaka 567‐0085 Japan; ^6^ RIKEN SPring‐8 Center Hyogo 679‐5148 Japan; ^7^ Department of Mechanical Engineering Manhattan College Riverdale NY 10471 USA; ^8^ Department of Environmental Health Harvard School of Public Health Boston MA 02115 USA; ^9^ Tsuda Lung Research Shrewsbury MA 01545 USA; ^10^ Nanoblesse Research Lab. Pohang 37883 South Korea; ^11^ Graduate School of Artificial Intelligence, POSTECH Pohang 37673 South Korea

**Keywords:** alveolar ducts, alveolus, gas exchange, lung volume, microdynamics, surface area, synchrotron

## Abstract

It is self‐evident that our chests expand and contract during breathing but, surprisingly, exactly how individual alveoli change shape over the respiratory cycle is still a matter of debate. Some argue that all the alveoli expand and contract rhythmically. Others claim that the lung volume change is due to groups of alveoli collapsing and reopening during ventilation. Although this question might seem to be an insignificant detail for healthy individuals, it might be a matter of life and death for patients with compromised lungs. Past analyses were based on static post‐mortem preparations primarily due to technological limitations, and therefore, by definition, incapable of providing dynamic information. In contrast, this study provides the first comprehensive dynamic data on how the shape of the alveoli changes, and, further, provides valuable insights into the optimal lung volume for efficient gas exchange. It is concluded that alveolar micro‐dynamics is nonlinear; and at medium lung volume, alveoli expand more than the ducts.

## Graphical abstract

Gas exchange is enhanced when the alveolar volume increases more than the alveolar duct's volume because an increase in alveolar volume increases the surface area available for gas exchange but the ducts do not directly contribute to gas exchange. This study reveals that more air enters the alveolus than the alveolar ducts, showing the highest alveolar wall compliance, in the case of medium lung volume.

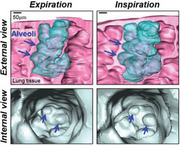

## 1 Introduction

To maintain life, oxygen (O_2_), an essential ingredient in the creation of energy in our cells, must be constantly provided to the body, and the gaseous metabolic waste, carbon dioxide (CO_2_), must be continuously expelled from the body. Since this gas exchange is performed in the lung parenchyma by diffusion, it is fundamental to know how efficiently the lung parenchyma are functioning; namely, how much lung surface is available for gas exchange during tidal ventilation relative to the increase of the lung volume. In other words, it is crucial to elucidate the relationship between parenchyma surface area (𝑆) and volume (𝑉) during tidal ventilation. While there may be some reserve in the case of healthy lungs, the available surface area for gas exchange could be on the edge of being insufficient in the case of diseased lungs. Therefore, it is crucially important to know the baseline of the relationship between 𝑆 and 𝑉 in order to diagnose the severity of diseases and to design strategies for their treatment. Specifically, efficient gas exchange requires a large surface area, and it is advantageous if the increase in alveolar surface area over inhalation is achieved with the smallest possible increase in alveolar pressure. For a given alveolar volume, minimizing the increase in pressure minimizes the work required to expand the surface area. Also, gas exchange is further enhanced if the air entering the alveolar space increases the volume of the alveoli (A) rather than the volume of the alveolar ducts (AD). In this report, we outline a new way of estimating the change in the distribution of surface area over the expanding volume of an alveolar unit (A and AD), and show that this new method can predict the division of air entering these two regions. While experts^[^ ^1^, ^2^ ^]^ in lung physiology recognize the importance of this knowledge, technical limitations have thus far rendered this information unobtainable. Moreover, to our surprise, we even lack sufficient data to determine whether gas exchange occurs primarily through the expansion of aerated alveoli or the recruitment of collapsed alveoli. This knowledge gap can be also attributed to previous technical constraints. However, the novel visualization technique presented in this study, which allows for the observation of alveolar microdynamics in live animals, brings us closer to establishing the essential baseline of alveolar microdynamics.

Traditionally, all measurements in microscale were made statically; the 𝑆–𝑉 relationship was deduced by making morphometric measurements of the alveolar system structure (e.g., alveolar size, ductal size, etc.) in 2D histologic samples fixed at various target volumes. However, these traditional approaches suffer significant artifacts, such as postmortem deformation, a lack of blood flow, or significant tissue shrinkage during the fixation process.^[^ ^3^, ^4^ ^]^

Recently, several new technologies, such as intravital microscopy (IVM) and optical coherence tomography (OCT), which observe alveolar microdynamics through a small window made on the pleural surface, have been developed to make measurements without fixation. However, even with these new approaches performed on living animals, they are not perfectly non‐invasive. They are, in fact, unphysiological, suffering serious shortcoming for highly sensitive/interdependent organs like lungs.^[^ ^5^, ^6^ ^]^ To achieve noninvasive measurement, synchrotron‐based X‐ray tomography has recently been used.^[^ ^7^, ^8^ ^]^ However, even in these studies, the visualization was unphysiological; the measurements were performed during breath holding to avoid the blurriness caused by cardiac motion. While these studies might be interesting for their purposes, this technique cannot be used to study morphological change of the alveolar system (i.e., alveoli and their associated alveolar ducts) during tidal ventilation. In fact, the authors concluded that the study of alveolar tidal dynamics by synchrotron tomography is impossible. In this study, we challenge this notion. In the field of physics,^[^ ^9^ ^]^ it is known that as one moves closer to the axis of twist, the magnitude of circumferential motion diminishes. Since motion artifacts caused by the heartbeat are predominantly influenced by left ventricular twist,^[^ ^10^, ^11^, ^12^, ^13^ ^]^ we focused on studying the alveoli situated in the apical region of the lung, which aligns with the axis of cardiac rotation. In this study, based on this physics principle, we have visualized the shape change of the alveolus during tidal ventilation without breath holding in a mouse model by synchronizing the camera with the respiratory cycle (explained further below).

To visualize the tidal ventilation dynamics of the 𝑆–𝑉 relationship of the alveolar system in live animals, the following three conditions must be met simultaneously. 1) The visualization has to be performed non‐invasively. Since there is a strong interdependence between various regions of the lungs (i.e., the local conditions can affect the conditions of other parts)^[^ ^5^ ^]^ and the structure of each individual alveolus is maintained by a delicate balance of various tensions, like a parachute,^[^ ^14^ ^]^ the natural structure and dynamics of an alveolus cannot be represented accurately if this delicate balance is disturbed due to invasive manipulations. Therefore, the motion of the alveolar system has to be visualized non‐invasively in vivo. 2) The resolution of the visualization is fine enough so that the structure of each alveolus can be imaged. The typical size of mouse alveoli is of the order of 50∼100 µm^[^ ^15^, ^16^, ^17^ ^]^ (cf. the size of human alveoli is of the order of 200 µm).^[^ ^18^ ^]^ Hence, at least one hundred times finer resolution would be necessary to visualize the detail of alveolar structure. 3) The speed of visualization should be much faster than the ventilation period, which is of an order of a second or less in the mouse case, to track the breathing dynamics. This also requires an extremely high photon flux for visualization. Currently, only a few synchrotron‐based X‐ray visualization systems in the world can meet these three conditions simultaneously. One such system is the SPring‐8 synchrotron (Hyogo, Japan), which we used for this experiment.

While we have previously reported the idea of tracking the dynamics of the lung parenchyma of a ventilated mouse in vivo,^[^ ^19^, ^20^ ^]^ those studies suffered from motion artifacts due to the rapid movement of the parenchyma during ventilation. In this study, we significantly reduced the influence of motion artifacts by switching the anesthesia system to maintain stabilized ventilation and by increasing the sampling rate by improving the triggering technique (explained in detail later).

The ultimate goal of this study is to increase our fundamental knowledge of the alveolar system mechanics. To achieve this goal, we need to study the dynamics of alveolar tidal behavior by directly visualizing the alveolar system in a living mouse during tidal ventilation. Alveolar size, and their wall thickness were measured in 3D volume render structures directly at the maximum and minimum lung volume at three different PEEP (positive end expiratory pressure) values (0, 3, 10 cm H_2_O). These 3 PEEPs correspond to low, medium, and high initial lung volumes, respectively. From the difference in alveolar surface area and perimeter of the alveolar opening between those two lung volumes during tidal ventilation, the strains of the alveolar membrane (*ε*_A_) and that of the alveolar entrance ring (*ε*_ER_) were measured and calculated. To quantify how the surface area for gas exchange changes relative to an increase of the lung volume at the alveolar level, we measured the surface area (𝑆) and volume (𝑉) of the alveolar system at those two lung volumes. By comparing the shape of the structure at these two extreme time points, the changes in the structural shape were determined during tidal ventilation. As mentioned above, it is fundamental and significant to determine the baseline in the healthy lung in order to diagnose the severity of abnormality and to construct strategies for the treatment of diseases of the lungs, which involve impairments of the parenchyma. We believe that the new visualization technique described in this paper brings us to the point where we can start to quantify alveolar micro‐dynamics in the healthy lung.

## 2 Results

**Figure 1:**
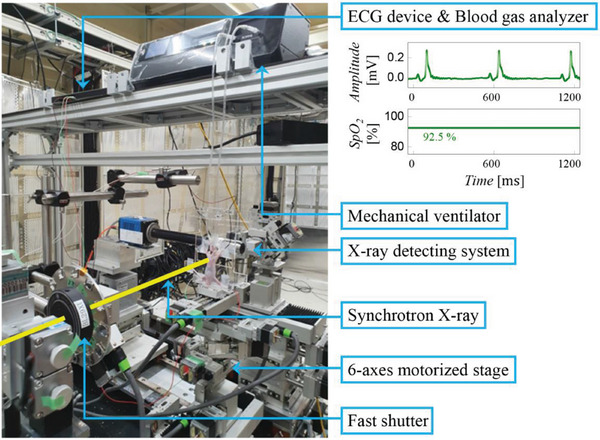
Photograph of X‐ray microtomography setup. Electrocardiogram (ECG) and Saturation pulse oxygen (SpO_2_) of the mouse are monitored in real‐time.

**Figure 2:**
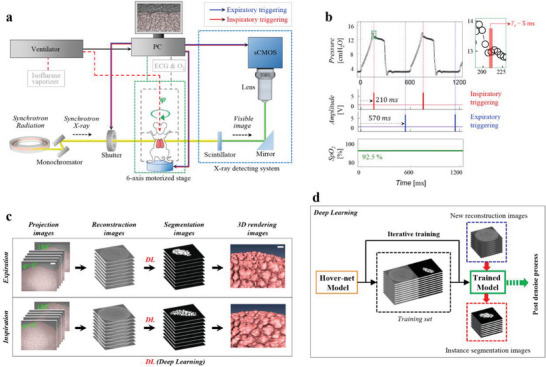
X‐ray microtomography setup and deep‐learning (DL) for automatic segmentation. a) Schematic of the microtomography setup. Synchrotron X‐ray (represented by the yellow thick line) is used to image the lung of a live anesthetized mouse. The mouse is positioned on a 6‐axis motor controller (indicated by the green dashed box) and undergoes ventilation during imaging. The X‐ray detection system (depicted by the blue dashed box) captures the images. The alive status of the animal was ensured by constant monitoring of blood gas oxygenation levels and ECG signals (shown as gray dashed lines). b) A CMOS camera was synchronized with respiration by triggering at two time points: inspiration (indicated by the red line) and expiration (represented by the blue line) during each ventilation cycle. The camera had a sampling rate of 200 Hz with a sampling duration of 5 ms/image, as shown by the open solid circles in the inset. The settling time of the transient response was 210 ms, thus, the camera was triggered for inspiration at 210 ms after the onset of inspiration. The blood gas oxygenation level, which is continuously monitored, is also presented. c) The overall data processing involves three sequential steps after image projection: reconstruction, segmentation, and 3D rendering. Scale bar: 200 µm. d) Deep‐learning is employed for automatic segmentation. The Hover‐net model (depicted by the yellow box) is trained iteratively by comparing predicted images with manually segmented images (indicated by the black dashed box). A new set of reconstruction images (represented by the blue dashed box) is then segmented using the trained model (depicted by the green box), resulting in instance segmentation images (indicated by the red dashed box). The resulting instance segmentation images undergo a post‐denoising process (shown by the green dotted arrow).

We conducted synchrotron‐based X‐ray microtomography, segmentation, and volume rendering to analyze the 3D structures of live mouse lungs at maximum and minimum tidal volumes under three different PEEP values (0, 3, 10 cm H_2_O). For each condition, the microtomography generated 2160 reconstructed images with a pixel size of 0.65 µm,^[^ ^22^ ^]^ and the resulting 3D volume rendered field had dimensions of 1664 × 1664 × 1404 µm (**Figures** **1** and **2**).

### 2.1 Alveolar Size

**Figure 3:**
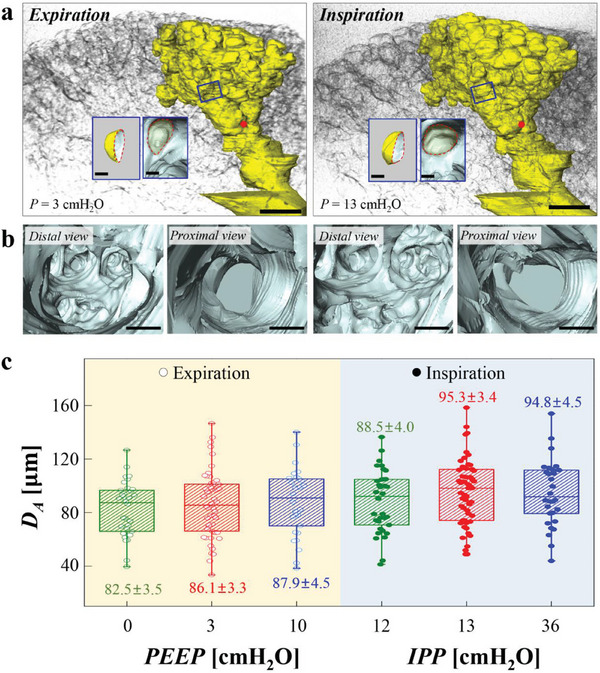
The acinar tree and the alveolar size of live mice. a) Representative views of the acinar tree at the minimal lung volume (left) and at the maximal lung volume (right) of a 3D volume rendered live mouse. Approximately one hundred alveoli are visible in the field. One representative acinar tree is highlighted in yellow. The size and shape of the tree visibly differ during ventilation, as shown in the left panel compared to the right panel at PEEP = 3 cm H_2_O. Scale bar = 200 µm. Inset: Appearances of representative alveoli. The red dotted curves show the alveolar entrances. Scale bar = 20 µm. b) Internal views from the area indicated by the red dot in panel (a). In the distal direction, numerous alveolar openings and an acinar bifurcation are observed. In the proximal direction, relatively smooth‐walled small airways are seen. Scale bar = 50 µm. c) Alveolar diameter (*D*_A_) versus three values of PEEP (0, 3, or 10 cm H_2_O) and of IPP (inspiratory plateau pressure) corresponding to the respective PEEPs. A total of 118 individual alveoli (34, 54, 30 in 3 mice for PEEP = 0 cm H_2_O, in 6 mice for PEEP = 3 cm H_2_O, in 3 mice for PEEP = 10 cm H_2_O, respectively) were measured. It is worth noting that it is hard to conceive that alveoli at 36 cm H_2_O have a median diameter that is less than that at 13 cm H_2_O. However, this may have been due to predominant inflation of deeper and more compliant alveoli, which receive a larger fraction of tidal breathing. The dataset is presented as a box‐and‐whisker plot, based on the five‐number summary, which includes the minimum, the maximum, the sample median, and the first and third quartiles.^[^ ^24^ ^]^ The colored numbers represent mean ± standard error values.

**Figure 4:**
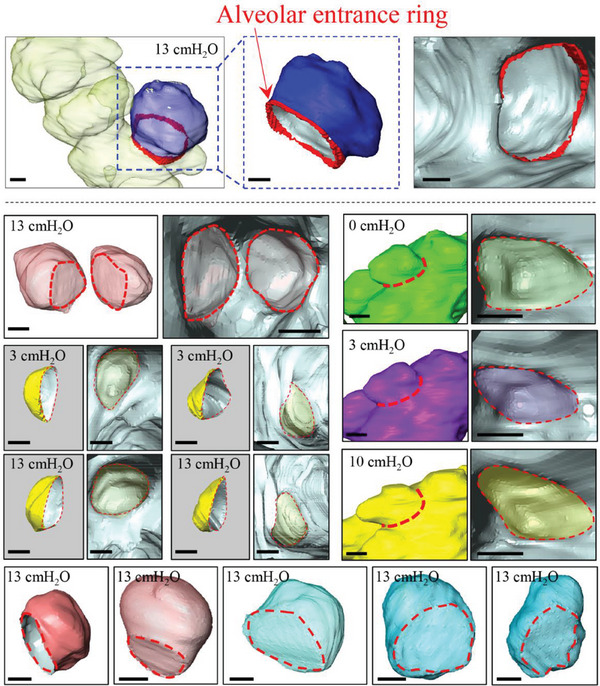
Examples of alveolar entrance ring. To isolate an alveolus from the alveolar duct, we determined a border between the alveolus and the alveolar duct (shown in red dot curves), which represents the aperture of the alveolus. Scale bar: 30 µm. 3 mice for PEEP = 0 cm H_2_O, 6 mice for PEEP = 3 cm H_2_O, and 3 mice for PEEP = 10 cm H_2_O were analyzed.

The representative images in **Figure** **3**a depict the minimal lung volume (left) and the maximal lung volume (right), displaying numerous (approximately 100) alveoli, with a highlighted acinar path shown in yellow. The insets in Figure 3a present typical alveolar appearances. To isolate an alveolus from the alveolar duct, we determined the border between them, known as the alveolar entrance ring (indicated by a red curve), by examining the internal view of the area. The location of the alveolar entrance ring was carefully identified based on the sudden appearance and disappearance of the alveolar wall (**Figure** **4**).^[^ ^23^ ^]^ The internal views in the distal direction from a chosen point (red dot in Figure 3a) reveal multiple alveolar openings forming the alveolar ducts and the acinar bifurcation. Conversely, the internal views in the proximal direction from the same point show relatively smooth‐walled small airways.

Figure 3c presents the apparent diameters of the alveoli (*D*_A_), which are the diameters of equivalent spherical alveoli, at three PEEP values (0, 3, 10 cm H_2_O) and three inspiratory plateau pressure (IPP) values (12, 13, 36 cm H_2_O) resulting from ventilation (i.e., a tidal volume of 10 mL kg^−1^ at 100 breaths per min). The apparent diameters, *D*_A_ = (6*V*/π)^1/3^ , are the diameters of equivalent spherical alveoli. The volumes, *V*, of 118 individual alveoli (34, 54, 30 alveoli for PEEP = 0, 3, 10 cm H_2_O cases, respectively) were calculated^[^ ^25^ ^]^ using the triangle patch function (where the alveolar surface and opening area were covered with triangles and the volume of the closed object was divided into tetrahedra) of Amira 5.2 software (Visage Imaging, USA). These measurements indicate that the live alveolus size, which is significantly larger than previously estimation based on the fixed samples,^[^ ^15^, ^16^, ^17^ ^]^ does not change linearly with the applied pressure, showing that the material characteristics of the alveolus change with the volume of the lungs. Notably, when the lung volume starts at PEEP = 3 cm H_2_O, the difference in alveolus size between expiration and inspiration is more than 50%, exceeding the differences observed in low (PEEP = 0 cm H_2_O) and high (PEEP = 10 cm H_2_O) lung volume cases by 30%. This suggests high sensitivity of alveolar wall compliance to PEEP values, and the potential advantages of medium PEEP for gas exchange. In the following analysis, we measured various micro parameters (e.g., *S*_A_, *T*_A_, *ε*_A_, and *ε*_ER_) during ventilation to gain further insights into alveolar microdynamics.

### 2.2 Tissue Characteristics

**Figure 5:**
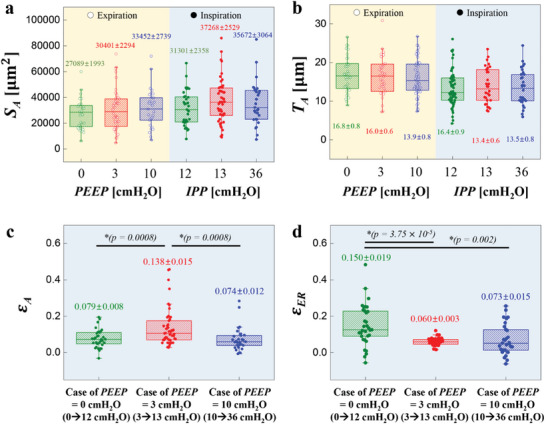
Alveolar stretchability. a) S_A_ versus PEEP & IPP. b) *T*_A_ versus PEEP & IPP. c) ε_A_ versus three cases of PEEP. d) ε_ER_ versus three cases of PEEP. 3 mice for PEEP = 0 cm H_2_O, 6 mice for PEEP = 3 cm H_2_O, and 3 mice for PEEP = 10 cm H_2_O.

Gas exchange through the alveolar wall occurs via diffusion, and the rate of diffusion is influenced by four factors. These factors include the surface area available for diffusion, the thickness of the air‐blood barrier, the difference in partial pressure of gases, and the tissue permeability. The first two factors are related to the structure of the alveoli. Hence, we measured the surface area (*S*_A_) and wall thickness^[^ ^26^ ^]^ (*T*_A_) of individual alveoli and examined their changes during tidal ventilation at three different PEEPs (**Figure** **5**a,b—also refer to Figure S4, Supporting Information).

The results demonstrate that the increase in alveolar surface area throughout the ventilation cycle is significantly larger at the mid‐lung volume (PEEP = 3 cm H_2_O) compared to low or high lung volumes (PEEP = 0 cm H_2_O or 10 cm H_2_O, respectively). Similarly, the thickness of the alveolar wall decreases the most during ventilation cycles at the mid‐lung‐volume cases (PEEP = 3 cm H_2_O). These findings suggest that more efficient gas exchange can be achieved at PEEP = 3 cm H_2_O (mid‐lung volume) compared to PEEP = 0 cm H_2_O (too low lung volume) or PEEP = 10 cm H_2_O (too high lung volume).

It is known that the compliance, or stretchability, of the alveolar wall differs from that of the entrance ring due to variations in tissue/fiber composition.^[^ ^27^, ^28^, ^29^ ^]^ Therefore, we divided the alveolus into two parts: the wall membrane area and the entrance ring. We measured the strain increase of each part with respect to PEEP (Figure 5c,d). To compare the strain of two parts, the area strain Δ*S* [ =  (*S*_Insp_ − *S*_Exp_)/*S*_Exp_] was converted to the linear strain ε [  =  (*L*_Insp_ − *L*_Exp_)/*L*_Exp_, where *L* represents the length scale] by using the relationship expressed as ε  = (1 + Δ*S*)^1/2^  − 1.^[^ ^6^ ^]^ As expected, alveolar wall strain (ε_A_) is substantially higher (more than double) than the entrance ring strain (ε_ER_) at PEEP = 3 cm H_2_O. It is further interesting to see that ε_A_ at PEEP = 3 cm H_2_O is substantially higher (nearly double) than ε_A_ at other PEEP values, suggesting that alveolar wall stretchability is highly influenced by the lung volume. It is equally interesting to see ε_A_ and ε_ER_ at PEEP = 10 cm H_2_O are nearly the same. This may suggest that the alveolar walls are overly stretched at the higher PEEP value so that the septal fiber system is already in a nearly‐fully‐extended state. The most unexpected finding is that ε_ER_ is larger than ε_A_ at PEEP = 0 cm H_2_O. A large variance in ε_ER_ at this PEEP may suggest that the alveolar structure, which is largely determined by the axial fiber system, is in an unstable balance between the alveolar fiber system and the pleural fiber system.

### 2.3 Alveolar Shape Changes during Ventilation

**Figure 6:**
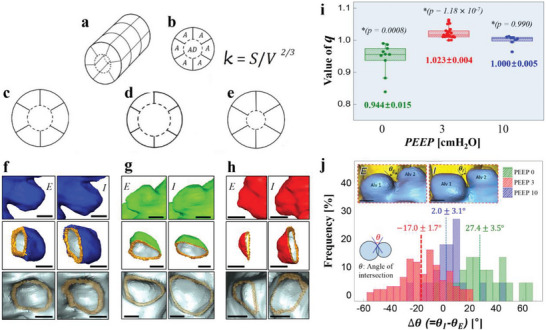
Shape changes of the structure during tidal ventilation. a) Schematic view of a gas‐exchange unit. b) Cross‐sectional view of a. c–e) Schematic views of three cases, *q* = 1, *q* < 1, or *q* > 1, respectively (see text for definition of *q*). f–h) Typical shape change of a representative alveolus for three cases (balloon‐like isotropic change for *q* = 1; widening aperture relative to alveolar depth for *q* < 1, deepen alveolus relative to the size change of alveolar opening for *q* > 1). i) *q* versus PEEP. j) The angle, θ, between two neighboring alveoli. At PEEP = 0 cm H_2_O, 22 sets of alveoli over 3 live mice, at PEEP = 3 cm H_2_O, 92 sets of alveoli over 6 live mice, at PEEP = 10 cm H_2_O, 22 sets of alveoli over 3 live mice were analyzed.

The lung parenchyma is comprised of groups of alveoli (A) clustered around a common space called “alveolar ducts (AD)” (depicted schematically in **Figure** **6**a–e). The hypothetical ductal “wall” is shown as a broken line in Figure 6a–e to emphasize that it is mostly composed of alveolar openings. Exchange of gases (i.e., O_2_, and CO_2_) with the blood in the capillary network primarily takes place at the alveolar surface with the role of the ducts being to transport air to and from the alveoli. Therefore, as the volume of a gas‐exchange unit (AAD = A + AD) increases over inhalation, the efficiency of the gas exchange is related to the division of the inhaled air between the alveoli and the alveolar ducts. Gas exchange would be more effective if the oxygen‐rich air mostly enters the alveoli to increase their surface area, rather than being used to increase the volume of the duct. Hence, we are interested in how the surface area is related to the volume of the gas exchange unit.

Since the dimension of the surface area (*S*) is the square of the length scale (*L*) and the dimension of the volume (*V*) is the cube of *L*, *S* is dimensionally always proportional to *V* ^2/3^, mathematically expressed as *S* ∝ *V* ^2/3^, or *S*  =  *kV* ^2/3^, where *k* is a dimensionless parameter.^[^ ^30^ ^]^ A higher value of *k* indicates a larger surface area relative to the volume. Therefore, a change in shape can be inferred from a change in *k*. We measured the volume and surface area of the gas exchange unit at the maximal and minimal lung volumes and defined the degree of shape change as *q*  = *k*_insp_/*k*_exp_.

If *q* = 1, the shape of the gas exchange unit at inspiration remains the same at expiration (Figure 6c,f). This implies an isotropic or geometrically similar change in structure, resembling the behavior of a balloon. Conversely, if *q* ≠ 1, the shape of the gas exchange unit at inspiration differs from its shape at expiration, indicating a non‐isotropic change. Specifically, if *q* > 1, the shape change leads to greater expansion of the alveoli compared to the alveolar duct (Figure 6e,h). On the other hand, if *q* < 1, the alveolar duct expands more than the alveoli (Figure 6d,g).

We observed a significant dependence of *q* on the initial lung volume, indicating the complex nature of alveolar microdynamics (Figure 6i). Consequently, the expansion dynamics of alveoli cannot be predicted solely from macroscopic measurements, such as the airway opening pressure at the beginning and end of inspiration. At low lung volume (PEEP = 0 cm H_2_O), *q* < 1 (= 0.944 ± 0.015) (the probability of *q* = 1 is negligible, *p* = 0.0008), indicating that the inhaled air primarily increased the volume of the alveolar ducts rather than the alveoli. At very high lung volume (PEEP = 10 cm H_2_O), where the lungs were already extensively expanded, *q* = 1 (= 1.000 ± 0.005) (the probability of *q* = 1 is extremely high, *p* = 0.99), suggesting behavior similar to that of a balloon. At medium lung volume (PEEP = 3 cm H_2_O), *q* > 1 (= 1.023 ± 0.004) (the probability of *q* = 1 is extremely low, *p* = 1.18 × 10^−7^), indicating non‐isotropic behavior with greater expansion of the alveoli compared to the alveolar duct.

To visually demonstrate the isotropic‐anisotropic behavior (Figure 6j), we measured the angle θ between two representative neighboring alveoli at PEEP = 0, 3, 10 cm H_2_O. Interestingly, at PEEP *=* 0 cm H_2_O, θ increased during tidal ventilation (i.e., Δθ = θ_Insp_ − θ_Exp_ = 27.4 ± 3.5° (mean ± s.e.m) > 0) (the probability of Δθ = 0° is extremely low, *p* = 1.17 × 10^−7^), while at PEEP = 3 cm H_2_O, θ decreased during tidal ventilation (i.e., Δθ = −17.0 ± 1.7° < 0) (the probability of Δθ = 0° is negligible, *p* = 2.74 × 10^−16^), confirming the change in shape described above. In contrast, at PEEP = 10 cm H_2_O, the increase (or decrease) in θ was practically zero, (Δθ = 2.0 ± 3.1°) (the probability of Δθ = 0° is high, *p* = 0.52), indicating isotropic behavior.

## 3 Discussion

Due to the predominant reliance on postmortem static tissue samples, our current understanding of alveolar morphology is limited, and a baseline for alveolar microdynamics in healthy lungs has yet to be established.^[^ ^31^ ^]^ This lack of baseline data hinders the accurate diagnosis of disease severity and the development of effective treatment strategies. Moreover, to our surprise, we even lack sufficient data to determine whether gas exchange occurs constantly throughout the respiratory cycle of aerated alveoli or the recruitment of collapsed alveoli. This knowledge gap can be attributed to previous technical constraints. However, the novel visualization technique presented in this study, which allows for the observation of alveolar microdynamics in live animals, brings us closer to establishing the essential baseline of alveolar microdynamics.

We conducted high‐resolution, noninvasive synchrotron‐based X‐ray microtomography on the apical lung region of a ventilated live mouse and measured various dynamic parameters of the lung parenchyma at minimal and maximal lung volumes during tidal ventilation. Our findings demonstrate that the microscale dynamic behaviors of the lung parenchyma exhibit high nonlinearity, challenging the notion that scaling macroscale measurements can capture these dynamics accurately. For instance, while it is commonly assumed that the compliance of the alveolus is significantly greater than that of the alveolar entrance area because the stiffness of the material composition of the alveolar membrane (e.g., epithelial cells, endothelial cells, capillaries, connective tissue, and septal fibers) and that of the alveolar entrance ring (e.g., containing the axial fibers) are very different, our new data revealed that such a notion is too simplistic; the reality is that alveolar compliance is largely influenced by lung volume.

One of the crucial factors determining efficient gas exchange is the relationship between the increase in alveolar surface area, where gas exchange occurs, and the amount of work exerted (i.e., a product of pressure and volume). Dimensional analysis shows that the relationship between the surface area (*S*) enclosing a volume (*V*) of any object can be expressed as S ∝ *V ^n^*, or *S* = *k V ^n^* where *n* = 2/3 and *k* is a dimensionless parameter.^[^ ^30^ ^]^ Past analysis^[^ ^1^ ^]^ committed a crucial error by assuming that *k* remains constant as the geometry's configuration changes and that *n* can have values other than 2/3 to indicate how the shape of the alveoli changes during ventilation. Recognizing this past mis‐conception, we have defined the ratio *q* = *k*_max_ */k*_min_ as a measure^[^ ^32^ ^]^ of how the shape of a gas‐exchange unit (a combination of the alveoli and their associated alveolar ducts) changes over ventilation.

Our findings reveal that *q* > 1 at the medium lung volume (PEEP = 3 cm H_2_O), indicating that a larger amount of air enters the alveoli rather than expanding the volume of the alveolar ducts. This observation aligns with our previous morphometric measurements, further supporting the notion of efficient gas exchange under these conditions.

Since our measurements are based on X‐ray imaging, it is important to address the topic of radiation dose. Recent reports on morphometric lung imaging^[^ ^33^, ^34^ ^]^ recommend to keep the X‐ray dose below 15 Gy based on the guidelines.^[^ ^35^, ^36^ ^]^ However, it is important to note that these guidelines, were primarily designed to minimize the long‐term cellular effects of high X‐ray doses used in cancer radiotherapy treatments. In our morphometric study, where the animals were sacrificed immediately after exposure, these classical guidelines are not directly applicable as we are primarily concerned with the short‐term effects of X‐ray dose on lung structure.

**Figure 7:**
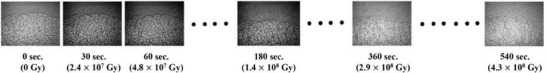
Radiation damage. By applying the X‐ray continuously, in the same manner as in the experiment but without interruption, the relationship between radiation dose and damage to the alveolar structure was monitored. There were no visible alterations in the structure within 3.6 s (calculated as 5 ms exposure duration for each imaging × 360 number of imaging for tomography × 2 for inspiration and expiration). In fact, structural alterations were not visible until 180 seconds.

We believe that the radiation‐induced structural damage in our experiment was negligible for several reasons. Firstly, all animals involved in the imaging experiment survived, indicating that the X‐ray dose was structurally within a safe range. Secondly, the dose administered during our experiment was several orders of magnitude lower than the dose known to cause visible structural damage (**Figure** **7**). Lastly, the successful performance of 3D volume rendering guarantees that the alveolar structure remained intact at the microscale, which would not have been possible if high radiation doses had caused significant structural changes.

**Figure 8:**
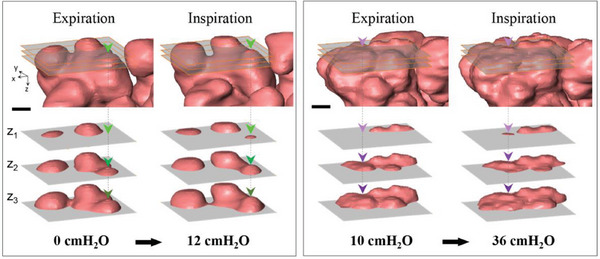
Recruitment/de‐recruitment. The sudden appearance of a new alveolus and alveolar clusters observed on optical imaging of subpleural alveoli under a coverslip after chest opening has been claimed as evidence of recruitment.^[^ ^40^ ^]^ However, alternative explanations can be proposed as follows. In PEEP = 0 cm H_2_O case (Left panel), the alveolus that appeared suddenly on the cut‐plane at *Z*_1_ during inspiration (12 cm H_2_O) (light green arrow head), could have actually been located just under the cut‐plane at *Z*_1_ in expiration. The same alveolus is shown as dark green arrowheads on the *Z*_2_ and *Z*_3_ cut‐planes in expiration. Similarly, in the PEEP = 10 cm H_2_O case (right panel), the alveolus that seemingly appeared suddenly on the cut‐plane at *Z*_1_ during inspiration (36 cm H_2_O) (indicated by the light violet arrowhead) could have actually been located just under the cut‐plane at *Z*_1_ in expiration [shown as dark violet arrowheads on the *Z*_2_ and *Z*_3_ cut‐planes during expiration (10 cm H_2_O)]. Therefore, these data suggest that the sudden appearance of a new alveolus and alveolar clusters on optical imaging of subpleural alveoli may not be evidence of recruitment.^[^ ^42^ ^]^ In fact, no recruitment was detected when we applied a very high lung pressure of 41 cm H_2_O, which is higher than that typically used for deep inflation in mice (≈35 cm H_2_O).^[^ ^43^ ^]^ Importantly, the thinning of elastic alveolar walls during inspiration (as observed in Figure 5 and Figure S1, Supporting Information) also provide evidence against the recruitment hypothesis, which assume negligible thinning of the walls during inspiration.^[^ ^6^, ^44^ ^]^

Although we cannot definitively dismiss the possibility of the recruitment/derecruitment^[^ ^37^ ^]^ mechanism due to the limited field of view in our study, we did not observe any indications of such a mechanism throughout our extensive experiments spanning over five years and involving hundreds of mice. This observation was supported by the previous studies.^[^^e^^.^^g^^.,^ ^38^ ^]^ The previous experimental evidence^[^ ^39^ ^]^ of recruitment,^[^ ^40^, ^41^ ^]^ based on the observation of sudden appearance of alveoli or alveolar clusters during inflation under a coverslip attached to the pleural surface through a small window of chest opening using in‐vivo optical microscopy, should be interpreted with caution^[^ ^42^ ^]^ (**Figure** **8**).

In this study, we have made several significant findings. First, we observed that the work required to deliver tidal air is strongly influenced by the initial lung volume, represented here by three different positive end‐expiratory pressures (PEEPs). This highlights the inadequacy of simple scaling laws to describe the changes in alveolar shape. The intricate dynamics of the alveoli cannot be predicted based solely on macro‐scale measurements. Furthermore, we discovered that the size of the gas‐exchange unit (AAD = A + AD) undergoes changes during ventilation, and the microdynamics of the alveoli exhibit nonlinear and complex behavior. Notably, we did not observe any instances of recruitment or de‐recruitment in our experiments. The change in shape of the alveolar system was highly sensitive to the lung volume. At low lung volumes, the expansion of the alveolar duct area surpassed that of the alveolar volume (consistence with the previous findings^[^ ^20^, ^45^ ^]^), indicating that a significant portion of inhaled ventilation gas is utilized indirectly for gas exchange by preventing the collapse of air passages rather than directly enhancing gas exchange. Conversely, at very high lung volumes, the alveolar system displayed geometrically similar changes in size. However, at this extreme lung volume, the distension of alveolar walls may have reached a point where further extension requires a considerably greater force (pressure). Interestingly, at a medium lung volume, the alveoli exhibited greater expansion compared to the alveolar duct, suggesting that the lungs are most efficient for gas exchange at this particular volume. Additionally, at this medium lung volume, the amount of work required to expand the lungs was found to be smaller compared to the other two volumes examined. Overall, these findings shed light on the intricate dynamics of the alveoli and provide valuable insights into the optimal lung volume for efficient gas exchange.

## 4 Experimental Section

### Animal Preparation

**Figure 9:**
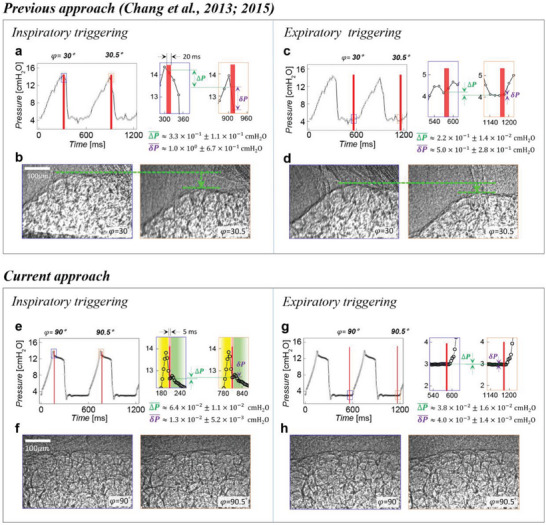
Minimalizing motion blurring artifacts in inspiratory and expiratory triggering. The difference in airway pressure between our previous studies and the current study has to be considered.^[^ ^19^, ^20^ ^]^ In both studies, there were two types of airway pressure differences: i) the pressure difference occurring during the short exposure time of 5 ms (note that it was 20 ms previously), δ*P* shown as purple arrows and ii) the pressure difference between breaths, Δ*P* shown as green arrows. Red bars denote the short exposure time of 5 ms (a– d). In our previous approach,^[^ ^19^, ^20^ ^]^ the large $\bar{\delta P}$ and $\bar{\Delta P}$ results in relatively large motion blurring artifact. a) Airway pressure wave of two consequent breaths during inspiration: $\bar{\delta P}\approx 1.0\times 10^{0}\pm 6.7\times 10^{-1}$ cm H_2_O, $\bar{\Delta P}\approx 3.3\times 10^{-1}\pm 1.1\times 10^{-1\;}\mathrm{cm}\;H2O$. b) The X‐ray image of the apex region corresponding to the airway pressure wave shown in (a). c) Airway pressure wave of two consequent breaths during expiration: $\bar{\delta P}\approx 5.0\times 10^{-1}\pm 2.8\times 10^{-1}$ cm H_2_O, and $\bar{\Delta P}\approx$ 2.2 × 10^−1^ ± 1.4 × 10^−2^ cm H_2_O). d) The X‐ray image of the apex region corresponding to the airway pressure wave shown in (c). e–h) Current approach. By keeping $\bar{\delta P}$ and $\bar{\Delta P}$ small, the blurring artifact can be kept negligible. e) Airway pressure wave of two consequent breaths during the inspiration: $\bar{\delta P}\approx$ 1.3 × 10^−2^ ± 5.2 × 10^−3^ cm H_2_O, $\bar{\Delta P}\approx 6.4\times 10^{-2}\pm 1.1\times 10^{-2}$ cm H_2_O. f) The X‐ray image of the apex region corresponding to the airway pressure wave shown in (e). g) Airway pressure wave of two consequent breaths during the expiration: $\bar{\delta P}\approx$ 4.0 × 10^−3^ ± 1.4 × 10^−3^ cm H_2_O, and $\bar{\Delta P}\approx 3.8\times 10^{-2}\pm 1.6\times 10^{-2}$ cm H_2_O). h) The X‐ray image of the apex region corresponding to the airway pressure wave shown in (g).

All experimental protocols were approved by the SPring‐8 Experimental Animals Care and Use Committee. Eight‐week‐old SPF pathogen‐free nude^[^ ^46^ ^]^ mice (BALB/c‐nu, body weight: 20–25 g, male, SLC Japan Inc., Japan) were anesthetized with an injection of a mixture of medetomidine, butorphanol, and midazolam, which put the animal into deep sleep in 10 min. A tracheostomy was performed, and a tightly secured 22 G Jelco I.V. catheter (Johnson & Johnson Medical, USA) was inserted. After surgery, the animal was immobilized in a vertical position in a custom‐made plastic holder (Figure 1) and placed in the experimental hutch for imaging. The catheter was connected to a mechanical ventilator (flexiVent, SCIREQ, Canada) (Figure 2a). Anesthesia was maintained by 2–3% of isoflurane in air/oxygen mixture through the catheter via the ventilator, significantly reducing pressure variation at every triggering point (**Figure** **9**e–h). The lung was ventilated in volume mode with the following ventilation parameters: tidal volume = 10 mL kg^−1^ of bodyweight; positive end‐expiratory pressure (PEEP) = 0, 3, 10 cm H_2_O; respiratory rate = 100 breaths/min.; I:E = 1:1.

### X‐Ray Microtomography of Live Lungs

X‐ray microtomography of live lungs was performed at the RIKEN Coherent X‐ray Optics beamline (BL29XUL) at SPring‐8 (http://www.spring8.or.jp), which provides the high spatial and temporal resolution required for this study, based on a bright monochromatic 15 keV X‐ray beam (6 × 10^13^ photons/s/mm^2^/mrad2/0.1%bw).^[^ ^47^ ^]^ The X‐ray beam was reduced by 30% due to X‐ray absorption through air. A schematic of the experimental setup is shown in Figure 2a, along with a photograph of the setup in Figure 1. A mouse, which was anesthetized and mechanically ventilated with a tidal volume of 10 mL kg^−1^ at 100 breaths min^−1^, was mounted on a 6‐axis motorized stage (Kohzu precision) using a custom‐made holder. The live status of the animal was ensured by continuously monitoring both blood gas oxygenation level and ECG‐signals (gray dashed line in Figure 2a). To minimize the influence of motion artifacts, an apical region aligned with the axis of cardiac rotation was selected as the field of view (FOV).^[^ ^10^, ^11^, ^12^, ^13^ ^]^ The synchrotron X‐ray (yellow thick line in Figure 2a) passing through the mouse was converted to a visible image (green thick line in Figure 2a using a scintillator crystal (LSO:Tb)). The image, after being reflected by a mirror and magnified by an optical lens (M Plan Apo HR 10×, Mitutoyo), was captured by an sCMOS camera (PCO.edge 5.5 CLHS, PCO AG, Germany) with a resolution of 2560 × 2160 pixels. The effective pixel size in this setup was 0.65 µm, and the exposure time was set to 5 ms.

### Synchronization with Respiration with a Camera Triggering at Inspiration and Expiration

For X‐ray microtomography, it is necessary to image the same region from a series of different angles ranging from 0° to 180°. Any drift of the object at the designated angle would render tomography impossible. To avoid blurriness caused by cardiac motion, some researchers synchronize the camera with the heartbeat and introduce breath‐holding. However, this approach does not achieve our goal. Therefore, instead, we synchronized the camera with the respiratory cycle to directly visualize alveolar dynamics.^[^ ^48^ ^]^ To minimize the influence of motion artifacts, we made several technical modifications. 1) The anesthesia system was switched from sodium pentobarbital, which was used in our previous studies^[^ ^19^, ^20^ ^]^ to a mixture of medetomidine, butorphanol, and midazolam. This change significantly stabilized the animal's conditions and ensured that the animal repeated the same motion during imaging (see Figure 9). Additionally, we continuously provided the animal with 2–3% isoflurane in an air/oxygen mixture through the endotracheal cannula via the mechanical ventilator (purple dashed line in Figure 2a). 2) The apical region was selected to image at both inspiration and expiration during each ventilation while rotating the animal at 0.5^○^ intervals. This specific area was chosen because motion is known to be minimal along the axis of rotation of an object^[^ ^9^ ^]^ and the apex aligns with the axis of cardiac rotation.^[^ ^10^, ^11^, ^12^, ^13^ ^]^ In addition to selecting the apical region as the field of view (FOV), a short inspiratory pause of 100 ms was imposed and we carefully examined the pressure waveform of the ventilation measured at the airway opening (Figures 2b and 9e,g) and observed the settling time of the transient response was 210 ms during inspiration of the ventilation in volume‐control mode (Figure 9e). Since this overshooting and settle phenomenon consistently occurred in every breath, The camera was triggered for inspiration at 210 ms after the onset of inspiration. A TTL (transistor‐transistor logic) signal was set to trigger the camera and the X‐ray shutter for the inspiration image (red line in Figures 2b and 9e). For expiration triggering (blue line in Figures 2b and 9g), the flat PEEP value region in the pressure waveform at 570 ms was selected after the onset of inspiration. All triggering control was performed using a self‐developed Visual Basic program (black arrow in Figure 2a). 3) Exposure time was significantly reduced from 20 to 5 ms by using a high numerical aperture (NA) lens (NA = 0.28,^[^ ^19^ ^]^ 0.42 in the current setup. 4) The sampling rate (to 200 Hz) compared to the original study was increased.^[^ ^19^, ^20^ ^]^ This reduction in exposure time and increasing in sampling rate made the breath‐by‐breath pressure variation (Δ*P*) very small and comparable to the pressure variability (δ*P*) that occurs during the 5 ms exposure duration (Figure 9e,g). Overall, the blurriness did not significantly affect the microtomography. The improvement was clearly evidenced by our ability to perform 3D volume rendering.

### Digital Signal Processing

In the microtomographic digital processing three sequential steps (reconstruction, segmentation, and 3D volume rendering) were involved after the projection of images and before the creation of the 3D structure (Figure 2c).

### Reconstruction

As the first step, the tomographic projection images were converted to segmentable 2D image datasets by retrieving the spatial information from the X‐ray raw images. Many commercial software packages are available for this step; the Octopus 8.9 software (Inside Matters NV, Belgium) was used. The examples of successful results of the reconstruction process are shown in Figure S1 and Video S1 (Supporting Information).

### Segmentation

A set of grayscale 2D reconstructed images was the input. Each pixel was assigns into a category. In the current case, every pixel was assigned to either tissue or air.

### 3D Volume Rendering

The final step, in which 3D structures were built from 2D segmented images. In the tomographic case, where the number of images to process is large, automatic segmentation was necessary. A deep learning (DL) approach was used in this study.

### Automatic Segmentation by Deep Learning

**Figure 10:**
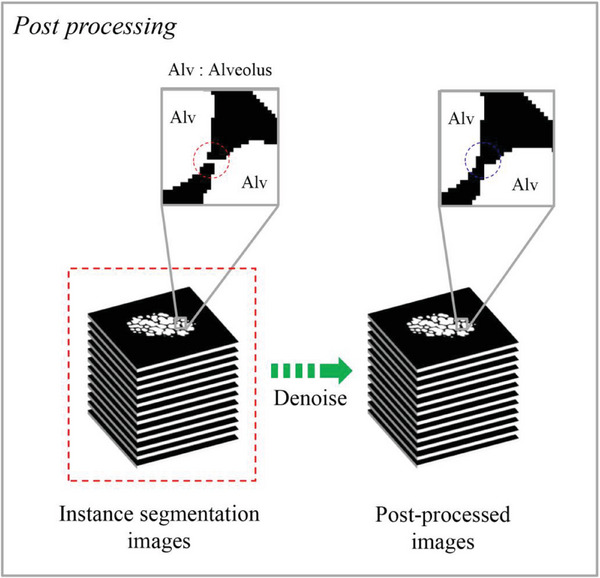
Minimalizing motion blurring artifacts in inspiratory and expiratory triggering. Unintended connection failures between two neighboring alveoli (red dashed circle) were repaired by the denoise process, resulting in proper separation of such two alveoli (blue dashed circle).

A schematic of the deep learning (*DL*) approach for automatic segmentation of reconstruction image sets is shown in Figure 2d. Hover‐net (yellow box in Figure 2d), which is well known as a segmentation neural network model,^[^ ^49^ ^]^ is first trained by learning optimal parameters, specifically by iteratively predicting segmentation images based on the reconstruction images (left panel of the training set‐black dashed box in Figure 2d) and comparing the predicted images to manually^[^ ^50^ ^]^ segmented images (right panel of the training set in Figure 2d) as a reference standard.^[^ ^51^ ^]^ Here, the trained model's validation accuracy is 0.9666, calculated by the mean intersection over union (mIoU),^[^ ^52^ ^]^ a method to quantify the percent overlap between target image and prediction output. The segmentation of a new set of reconstruction images (blue dashed box in Figure 2d) is then predicted (red arrow) by using the trained model (green box in Figure 2d), leading to the instance segmentation images (red dashed box in Figure 2d). Finally, the resulting segmentation images were denoised by a post denoise process (green dashed arrow in Figure 2d, also see **Figure** **10**).^[^ ^53^ ^]^ Specifically, unintended connection failures, as seen in the unintended connection between two neighboring alveoli (red dashed circle in the left inset of Figure 10), are repaired by the denoise process, resulting in proper separation of such two alveoli (blue dashed circle in the right inset of Figure 10). Figure S2 and Video S2 (Supporting Information) demonstrate the successful automatic segmentation based on the DL approach in expiration and inspiration.

The DL‐based automatic segmentation successfully enables 3D rendering using the Amira 5.2 software (Visage Imaging, USA) for both expiration and inspiration, as demonstrated in Figure 2c and Videos S3 and S4 (Supporting Information). The lung depth achieved in this study (≈1000 µm from the pleural surface) was more than four times that of previous studies and was large enough for 3D morphological analysis of alveoli clusters of significant size (Videos S3 and S4, Supporting Information). Previous studies of real‐time direct imaging of alveoli; such as, OCT,^[^ ^42^ ^]^ IVM,^[^ ^40^, ^42^ ^]^ and catheter‐based confocal microscopy,^[^ ^41^ ^]^ were limited to depths of less than 50 µm due to the use of visible light.

### Morphometric Measurements

Morphometric parameters of alveoli and alveolar ducts, delineated by 3D volume‐rendered images, such as the diameter and wall thickness of each alveolus, its strain, the surface area and volume of individual alveoli (A) and associated alveolar ducts (AD), and the angle of intersection between two neighboring alveoli (θ) were measured by using the Amira 5.2 software. Raw data are provided in Tables S1–S6 in Supporting Information.

### Diameter and Wall Thickness of an Alveolus

First, a volume (*V*) of the alveolus was calculated using the surface triangulation method.^[^ ^54^ ^]^ The diameter (*D*_A_) of an alveolus was calculated from the *V* assuming a spherical shape ($D_{A}=\sqrt[{3}]{\frac{6V}{\pi }}\;$). Then, the thinnest wall thickness was defined as *T*_A_ (green arrows in Figure S3, Supporting Information), and was measured in the 3D volume‐rendered structure.

(1) $$\mathrm{Surface}\;\mathrm{area}\;\mathrm{and}\;\mathrm{volume}\;\mathrm{of}\;\mathrm{AAD}\;\left(\;=\;A\;+\;\mathrm{AD}\right)$$

They were measured in the 3D volume‐rendered structure using Amira 5.2 software.

### Strains

The area strain Δ*S* [=  (*S*_Insp_ − *S*_Exp_)/*S*_Exp_] was converted to the linear strain ε [=  (*L*_Insp_ − *L*_Exp_)/*L*_Exp_, where *L* represents the length scale] by using the relationship expressed as ε  = (1 + Δ*S*)^1/2^  − 1.^[^ ^6^ ^]^

### Angle of Interaction

The angle of intersection between two neighboring alveoli (θ) was measured in the 3D volume‐rendered structure using the “3D angle” function.

### Statistical Analysis

Data analysis was performed using XLstat (Addinsoft, New York, NY) add‐in for Microsoft Excel. The statistical analysis was based on measurements in at least three different mice unless otherwise noted. The numerical values in Figures 3, 5, and 6i and Figure S4 (Supporting Information) were denoted as mean ± one standard error. For Figures 3c and 5, and Figure S4 (Supporting Information), statistical significance is determined by ANOVA. As a post‐hoc analysis, pairwise comparisons were performed using unpaired Student's t test for samples of unequal variances to calculate statistical significance. For Figure 6i, the difference of the “*q*” value from 1 and the Δθ value from 0° were analyzed by a one‐sample Student's *t*‐test.

## Conflict of Interest

The authors declare no conflict of interest.

## Author Contributions

M.W.K., S.Y., and U.Y. contributed equally to this work. Y.I., A.T., and J.H.J. conceived and designed the research. M.W.K., U.Y., R.N., H.C., N.K., M.Y., N.K., S.L., Y.I., A.T., and J.H.J. performed experimentation. S.Y. and H.H. performed computation. Y.I., Y.K., H.H., A.T., and J.H.J. supervised the research. Surface‐to‐volume analysis was performed by F.S.H. and A.T. A.T. structured the manuscript, M.W.K., J.H.J., and A.T. wrote the original draft, F.S.H. and Y.K. edited, and all authors reviewed the manuscript.

## Supporting information

Supporting Information

Supplemental Video 1

Supplemental Video 2

Supplemental Video 3

Supplemental Video 4

Supplemental Video 5

Supplemental Video 6

## Data Availability

The data that support the findings of this study are available in the supplementary material of this article.
