# Supplementary material for: Alveolar Microdynamics during Tidal Ventilation in Live Animals Imaged by SPring‐8 Synchrotron
Source: Adv Sci (Weinh). 2024 Jul 3;11(33):2306256. doi: 10.1002/advs.202306256 (PMC11434049; doi:10.1002/advs.202306256)
Supplement: Supplementary file 1 — Supporting Information [file ADVS-11-2306256-s001.docx]

Supporting Information

Alveolar micro-dynamics during tidal ventilation in live animals imaged by SPring-8 synchrotron

Min Woo Kim, Seung Hyeon Yu, Un Yang, Ryota Nukiwa, Hyeon Jung Cho, Nam Seop Kwon, Moon Jung Yong, Nam Ho Kim, Sang Hyeon Lee, Jun Ho Lee, Jae Hong Lim, Yoshiki Kohmura, Tatsuya Ishikawa, Frank S. Henry, Yumiko Imai, Seung Soo Oh, Hyung Ju Hwang, Akira Tsuda^*^, and Jung Ho Je^*^

Five figures (Figure S1-S5) and six videos (Video S1-S6), along with potential limitations of our study, and six raw data tables (Table S1-S6), are presented below.


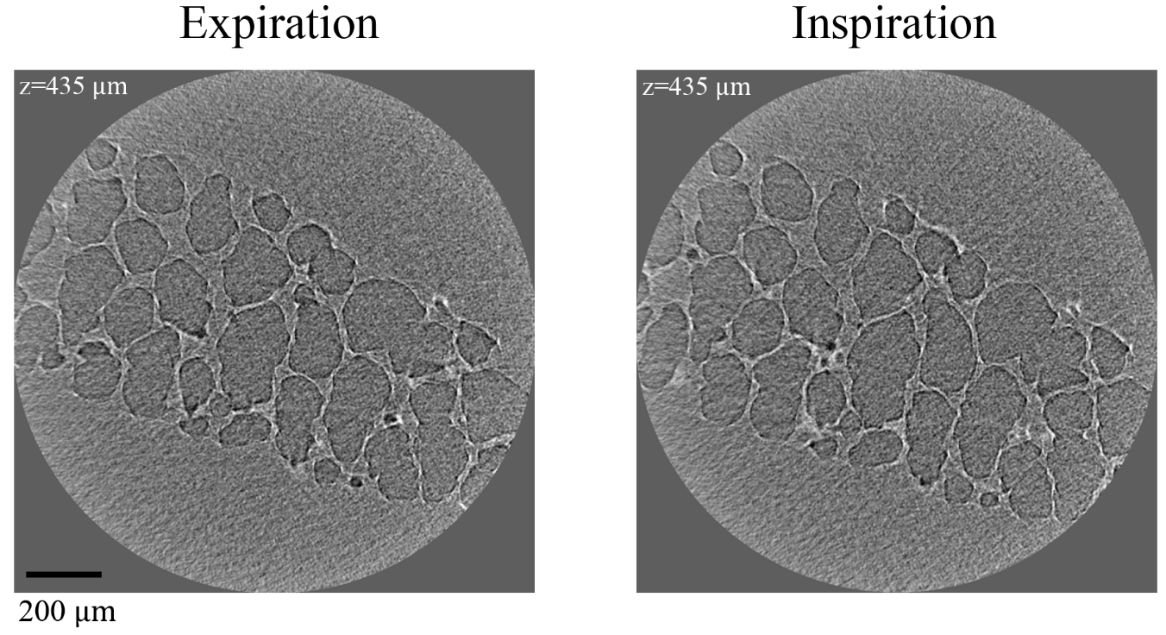


**Figure S1. Reconstructed image sets successfully retrieved from the projection image sets at expiration and inspiration using a commercial software.** Here, the z-axis displays the vertical direction from the top.


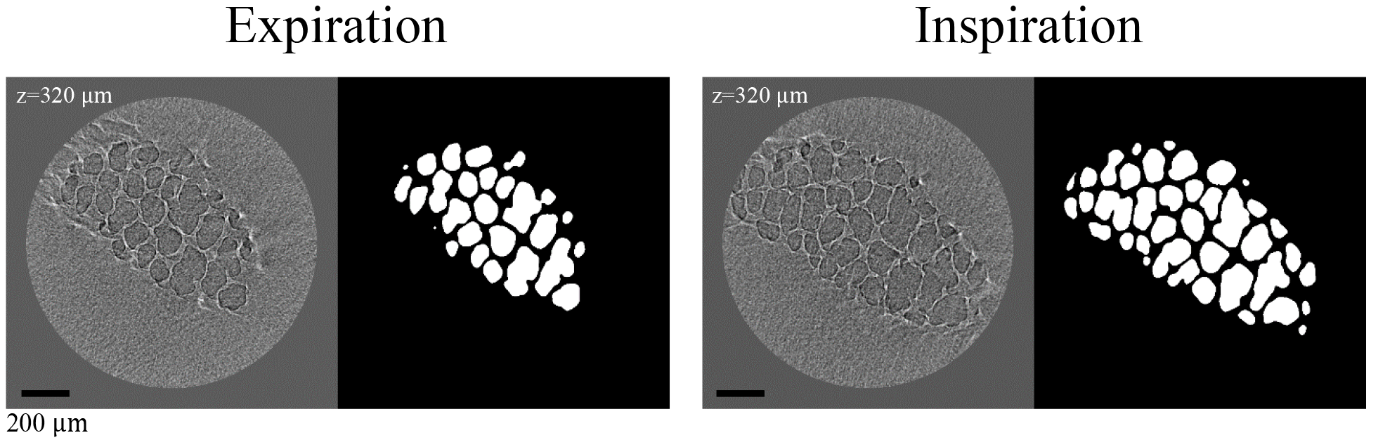


**Figure S2. Successful automatic segmentation (right) of reconstruction slice images (left) at expiration and inspiration by deep learning approach.**


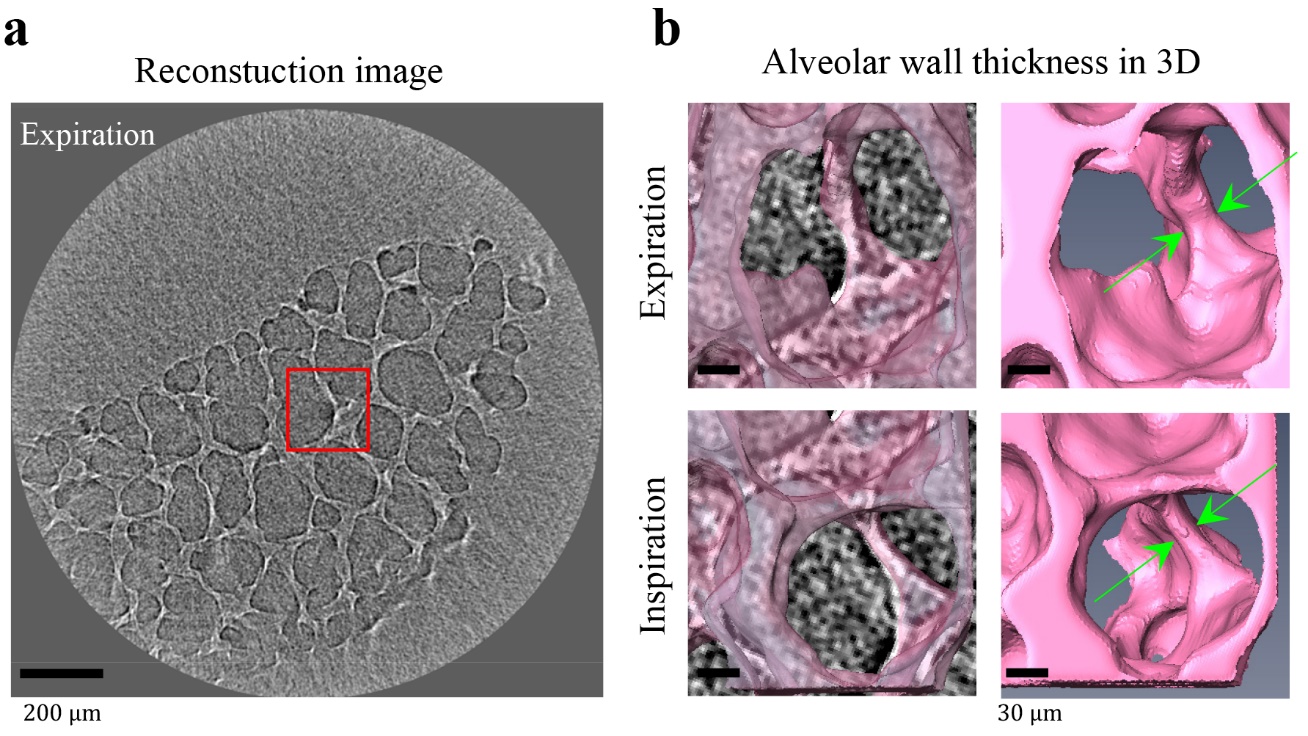


**Figure S3.** **Measurement of wall thickness of individual alveoli during respiration in live lungs. *a***: Reconstruction image of a live mouse lung at expiration. ***b***: 3D rendering images of the red box region of ***a*** at expiration (upper) and inspiration (lower). The thinnest wall thickness can be easily recognized (green arrows) and measured in the 3D rendering images.


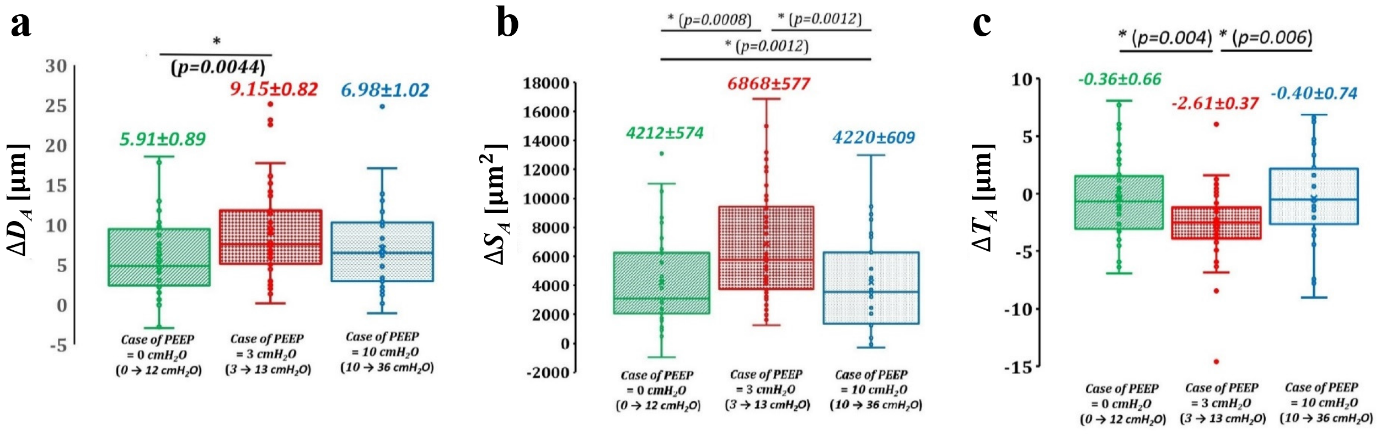


**Figure S4.** **Material characteristics of the alveolus changes with the volume of the lungs.** ***a*:** The size difference of the alveolus between expiration vs. inspiration ($\Delta$*D_A_*) did not change linearly with the applied pressure***.*** For instance, when the lung volume starts at PEEP = 3 cmH_2_O*,* $\Delta$*D_A_* is more than 50% and 30% larger than the cases of low (PEEP = 0 cmH_2_O) and high (PEEP = 10 cmH_2_O) lung volume, respectively. This suggests that the stretchability of the alveolar wall is highly sensitive to PEEP values. ***b***: The increase in alveolar surface area over a ventilation cycle was substantially larger at the mid-lung volume (i.e. at PEEP = 3 cmH_2_O) compared to the low or high lung volumes (at PEEP = 0 cmH_2_O or 10 cmH_2_O, respectively). ***c***: The thickness of alveolar wall reduced the most during ventilation cycles in the case of the mid-lung volume (i.e. at PEEP = 3 cmH_2_O).

The relationship between the alveolar surface area (*S*) versus the alveolar system volume (*V*)

Greaves et al. (1986)^[1]^ proposed a functional relationship between the growth of the alveolar surface area ($S$) and the alveolar system volume ($V)$; i.e., $S=kV^{n}$. Unfortunately, this paper contains a conceptual error. Greaves et al. (1986) assumed that the value of $k$ remains constant as the alveolar volume configuration changes. In the following, we explain why this assumption is incorrect.

As stated above, the relationship between the alveolar surface area (*S*) and the alveolar system volume (*V*) may be expressed as a power function, such as $S\propto V^{n}$ or $S=kV^{n}$. Taking logarithms of both sides of the equation, the power function equation $S=kV^{n}$ can be converted to an equation linear in ln *S* and ln *V*; i.e., ln *S* = ln *k* + *n* ln *V*. Therefore, *n* could be obtained morphologically as the tangent to the curve, i.e., local slope of the curve, by plotting ln *S* vs. ln *V*.

$n=\frac{d\left( \ln S \right)}{d\left( \ln V \right)}-\frac{d\left( \ln k \right)}{d\left( \ln V \right)}$ SI.1

In the case of two levels of expansion (i.e., two data points, measurements at inspiration and expiration. Note that this is the case in the present study.), Eq. SI.1 is expressed as

$n=\frac{\left( \ln S_{ins}-\ln S_{exo} \right)}{\left( \ln V_{ins}-\ln V_{exo} \right)}-\frac{\left( \ln k_{ins}-\ln k_{exo} \right)}{\left( \ln V_{ins}-\ln V_{exo} \right)}$ SI.2

Indeed, Greaves et al. (1986) describe how to obtain *n* experimentally by stating, “…was found by curve fitting in the expression S = *k*V*^n^*, where *k* and *n* are constants…..”. However, this statement is in error because *k* is only constant if the geometry expands in a geometrically similar fashion (i.e., an isotropic expansion). Therefore, in general, the second term of the right-hand side of Eq. SI.1 (or Eq. SI.2) is not zero.

In addition, in the analysis of Greaves et al. (1986) there is some ambiguity as to whether *k* is dimensionless or not. If *n* were not equal to 2/3, then *k* would not be dimensionless – it would have dimensions of length to the power of $2-3n$, for the left-hand side and the right-hand side of the equation to be dimensionally consistent. It seems implausible that $k$ would be dimensionless for one type of volume expansion (i.e., isotropic expansion) but have dimensions for all other types of expansion (i.e., anisotropic expansions).

The examples below are to support the fact that *n* is 2/3 even for anisotropic expansions and $k$ is a dimensionless variable that describes how the surface area changes with a change in the volume’s configuration.

Example 1

Consider an admittedly non-physical case in which an alveolus changes its shape from a cube of edge length $a$ at the end of expiration to a sphere of radius of $b$ at the end of inspiration.

Volume of a cube of side $a$

$$V=a^{3} \left( \text{SI.E1-1} \right)$$

Hence,

$$a=V^{1/3} \left( \text{SI.E1-2} \right)$$

Surface area of a cube of side $a$

$$S=6a^{2}=6\left( V^{1/3} \right)^{2}=k_{c}V^{2/3} \left( \text{SI.E1-3} \right)$$

where, $k_{c}=6$.

Volume of a sphere of radius $b$

$$V=\frac{4}{3}\pi b^{3} \left( \text{SI.E1-}\text{4} \right)$$

Hence

$$b=\left( \frac{3}{4\pi} \right)^{1/3}V^{1/3} \left( \text{SI.E1-}\text{5} \right)$$

Surface area of a sphere of radius $b$

$$S=4\pi b^{2}=4\pi\left[ \left( \frac{3}{4\pi} \right)^{1/3}V^{1/3} \right]^{2}=k_{s}V^{2/3} \left( \text{SI.E1-6} \right)$$

where, ${k_{s}=4\pi\left( 3/{4\pi} \right)}^{2/3}\approx4.836$.

We note that in both cases (shown in Eq.SI.E1-3 and Eq.SI.E1-6), $n=2/3$, and that $k$ is not constant ($k_{ins}=k_{s}\approx4.836$ and $k_{exp}=k_{c}=6$) during respiration but it is dimensionless.

Example 2: An anisotropic model of an alveolus: A cylinder with a fixed radius of $a$ increases its volume only by elongating its height

Volume of a cylinder of radius $a$ and height $h$

Let $h=\beta a$

$$V=\pi a^{2}h=\pi\beta a^{3} \left( \text{SI.E}\text{2}\text{-1} \right)$$

Hence,

$$a=\left( \frac{V}{\pi\beta} \right)^{1/3} \left( \text{SI.E}\text{2}\text{-}\text{2} \right)$$

Surface area of a cylinder of radius $a$ and height $h$

$$S=2\pi a^{2}+2\pi ah=2\pi\left( 1+\beta\right)a^{2}=2\pi\left( 1+\beta\right)\left( \left( \frac{V}{\pi\beta} \right)^{1/3} \right)^{2}$$

or

$$S=\frac{2\pi\left( 1+\beta\right)}{\left( \pi\beta\right)^{2/3}}V^{2/3} \left( \text{SI.E}\text{2}\text{-3} \right)$$

Hence

$$k=\frac{2\pi\left( 1+\beta\right)}{\left( \pi\beta\right)^{2/3}} \left( \text{SI.E}\text{2}\text{-4} \right)$$

We see that *k* is dimensionless & a function of *β* = *h*/*a* (i.e., not constant) and $n=2/3$.

Example 3: The Saucer-to-cup model of a ring of alveoli

The volume $V$ of the alveolus and the central channel shown in Figure S5 is

$$V=a^{3}\left[ \frac{4\pi}{3}+\frac{\pi^{2}}{2}d_{i}^{'}+\frac{\pi}{2}\left( d_{i}^{'} \right)^{2}+\left( \pi^{2}+2\pi d_{i}^{'} \right)h^{'}+2\pi\left( h^{'} \right)^{2} \right] \left( \text{SI.E}\text{3}\text{-1} \right)$$

where $h^{'}=h/a$, and $d_{i}^{'}={d_{i}}/a$. We note that for this expansion $d_{i}^{'}$ is fixed and thus the changes in volume are only as a function of $h^{'}$. Hence, we can write

$$V=a^{3}f\left( h^{'} \right) \left( \text{SI.E}\text{3}\text{-}\text{2} \right)$$

Hence

$$a=\frac{V^{1/3}}{\left[ f\left( h^{'} \right) \right]^{1/3}} \left( \text{SI.E}\text{3}\text{-}\text{3} \right)$$

The surface area $S$ of the alveolus shown in Figure S5 is

$$S=a^{2}\left[ 4\pi+\pi^{2}d_{i}^{'}+2\pi\left( \pi+d_{i}^{'} \right)h^{'}+2\pi\left( h^{'} \right)^{2} \right] \left( \text{SI.E}\text{3}\text{-}\text{4} \right)$$

We see that $S$ is also only a function of $h^{'}$. Hence, we can write

$$S=a^{2} g\left( h^{'} \right)=\left( \frac{V^{1/3}}{\left[ f\left( h^{'} \right) \right]^{1/3}} \right)^{2}g\left( h^{'} \right)=\frac{g\left( h^{'} \right)}{\left[ f\left( h^{'} \right) \right]^{2/3}}V^{2/3} \left( \text{SI.E}\text{3}\text{-}\text{5} \right)$$

We see that

$$k=\frac{g\left( h^{'} \right)}{\left[ f\left( h^{'} \right) \right]^{2/3}}=\frac{4\pi+\pi^{2}d_{i}^{'}+2\pi\left( \pi+d_{i}^{'} \right)h^{'}+2\pi\left( h^{'} \right)^{2}}{\left[ \frac{4\pi}{3}+\frac{\pi^{2}}{2}d_{i}^{'}+\frac{\pi}{2}\left( d_{i}^{'} \right)^{2}+\left( \pi^{2}+2\pi d_{i}^{'} \right)h^{'}+2\pi\left( h^{'} \right)^{2}f\left( h^{'} \right) \right]^{2/3}} \left( \text{SI.E}\text{3}\text{-}\text{6} \right)$$

That is, $k$ is dimensionless & only a function of $h^{'}$ (i.e., not constant) and $n=2/3$.


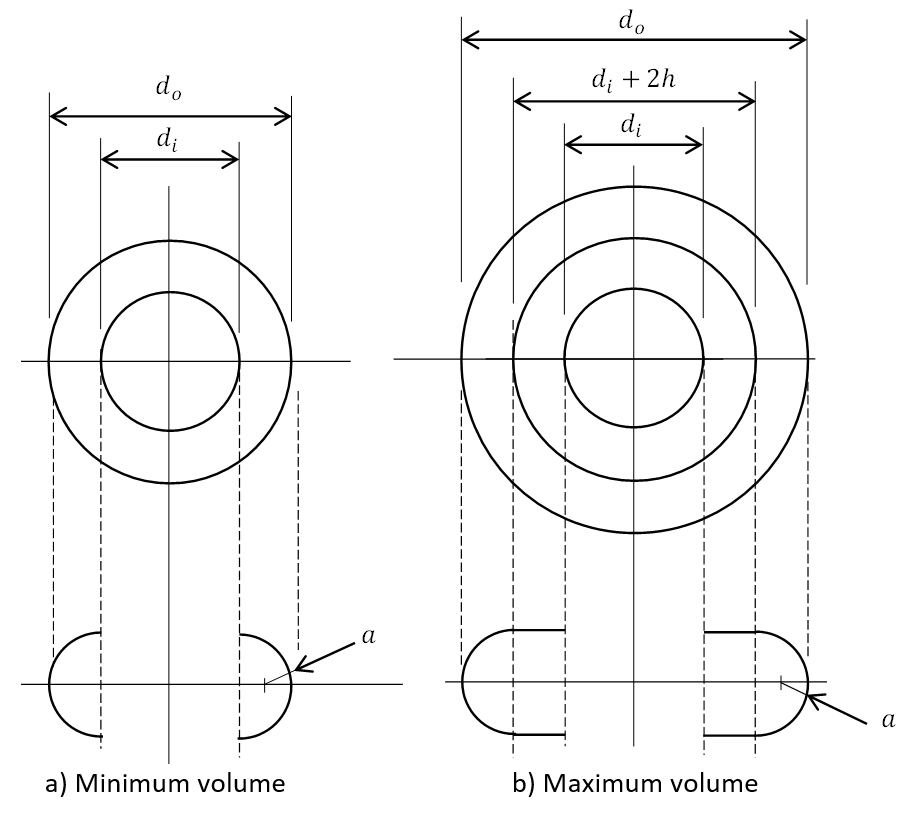


**Figure S5. Saucer to cup model of an alveolus.**

*A list of supporting videos*

**• Video S1:** Reconstructed image sets successfully retrieved from the projection image sets at expiration (left) and inspiration (right) using a commercial software. In the video, the z-axis displays the vertical direction from the top.

**• Video S2:** Successful automatic segmentation of reconstruction slice images at expiration (left) and inspiration (right) by deep learning approach. In the video, the z-axis displays the vertical direction from the top.

**• Video S3:** Successful volume rendering at inspiration.

**• Video S4:** Successful volume rendering at expiration.

**• Video S5:** 3D rendering images of eight alveoli and alveolar ducts (AAD) at inspiration.

**• Video S6:** 3D rendering images of eight alveoli and alveolar ducts (AAD) at expiration.

*A list of some of the potential limitations of our study*

Despite the current rapid technical advancement (e.g., computer power), our experiments have some potential limitations. They are listed below.

• **Field of view (FOV)**: One of the main limitations of our study is that the field of view (FOV) is small (~100 alveoli) compared to the size of the whole lungs. Because of the difference in scale between the size of the alveoli and that of the whole lungs, the number of images required to visualize the whole lungs at alveolar scale would be astronomical (We believe that this problem would eventually be solved in future with brute force by the increasing computer power). Although our size of FOV is limited, we believe that our study represents the beginning of alveolar micro-dynamical research.

• **Alveolar location (Apex)**: One may also wonder whether our alveolar measurements suffer a directional-biased expansion since the global view shows that the narrow apical space mainly expands vertically due to the rib cage restricting sideways motion. However, our measurements of core alveoli (~1000 $\mu m$ deep from the pleural surface) did not show such direction biased expansion.

• **2 time points**: We should mention that our dynamical information is based on measurements at 2 extreme time points in the cycle; namely, at the end of inspiration and at the end of expiration. First, we would like to emphasize that at least 2 time points are necessary to study dynamics. Second, although the fine viscoelastic nature of tissue characteristics may not be studied in detail from two discrete time points, such a topic is beyond a scope of this study.

• **Animal orientation**: To render the structure in 3D (tomography) the same region was imaged from different angles. While, in a regular hospital CT scan setting, the object (e.g., a patient) remains still and the machine rotates around the object, we had to do the opposite, namely, the machine remains still and the object rotates. This is due to the size of the machine (i.e., 1436m in a diameter). While there may be a concern that the unnatural orientation of the animal (upright position shown in Figure S1) may result in some non-physiological gravitational effects we believe this experimental setup is valid since gravitational effects are supposed not to be significant in small lungs and our measurements appears to be negligibly affected by the orientation of the animal.

*Raw data*

Below raw data are listed below.

**Table S1.** **Raw data of diameters of individual alveoli (**$\boldsymbol{D}_{\boldsymbol{A}}$**) in Figure 2c.** Diameters of individual alveoli ($\mu m$) in live lungs (3 mice for PEEP = 0 cmH_2_O; 6 mice for PEEP = 3 cmH_2_O; 3 mice for PEEP = 10 cmH_2_O).

| **PEEP = 0 [cmH_2_O]** | | | | **PEEP = 3 [cmH_2_O]** | | | | **PEEP = 10 [cmH_2_O]** | | | |
| --- | --- | --- | --- | --- | --- | --- | --- | --- | --- | --- | --- |
| Mouse No. | $D_{A\_Exp}$ [$\mu m$] | $D_{A\_Insp}$ [$\mu m$] | ${\Delta D}_{A}$ [$\mu m$] | Mouse No. | $D_{A\_Exp}$ [$\mu m$] | $D_{A\_Insp}$ [$\mu m$] | ${\Delta D}_{A}$ [$\mu m$] | Mouse No. | $D_{A\_Exp}$ [$\mu m$] | $D_{A\_Insp}$ [$\mu m$] | ${\Delta D}_{A}$ [$\mu m$] |
| #1 | 98.2 | 104.6 | 6.4 | #4 | 102.1 | 106.5 | 4.4 | #1 | 106.4 | 112.5 | 6.1 |
| #1 | 114.1 | 126.2 | 12.1 | #4 | 86.8 | 92.2 | 5.4 | #1 | 130.6 | 135.3 | 4.8 |
| #1 | 104.8 | 115.1 | 10.3 | #4 | 106.9 | 112.7 | 5.9 | #1 | 110.4 | 113.3 | 2.9 |
| #1 | 74.6 | 78.9 | 4.3 | #4 | 102.4 | 113.8 | 11.3 | #1 | 78.1 | 79.8 | 1.7 |
| #1 | 90.4 | 100.3 | 10.0 | #4 | 66.1 | 68.3 | 2.2 | #1 | 98.6 | 104.9 | 6.3 |
| #1 | 68.2 | 76.1 | 7.9 | #4 | 60.9 | 69.1 | 8.2 | #1 | 80.4 | 79.3 | -1.1 |
| #1 | 61.4 | 66.4 | 4.9 | #4 | 136.1 | 139.4 | 3.3 | #1 | 64.7 | 68.3 | 3.6 |
| #1 | 76.0 | 76.0 | 0.0 | #4 | 49.4 | 54.7 | 5.4 | #1 | 80.9 | 88.2 | 7.3 |
| #1 | 91.0 | 94.1 | 3.1 | #5 | 65.1 | 72.2 | 7.1 | #1 | 98.3 | 99.5 | 1.2 |
| #1 | 63.6 | 67.8 | 4.3 | #5 | 71.3 | 81.6 | 10.3 | #1 | 78.4 | 88.3 | 9.9 |
| #1 | 106.8 | 118.5 | 11.8 | #5 | 99.9 | 116.0 | 16.1 | #1 | 117.3 | 127.8 | 10.6 |
| #1 | 64.4 | 61.5 | -2.9 | #5 | 98.6 | 114.1 | 15.5 | #1 | 70.0 | 73.4 | 3.3 |
| #1 | 107.2 | 112.5 | 5.3 | #5 | 83.9 | 93.2 | 9.4 | #1 | 109.2 | 111.4 | 2.2 |
| #1 | 70.5 | 73.5 | 3.0 | #5 | 77.8 | 102.9 | 25.1 | #1 | 82.2 | 89.4 | 7.2 |
| #2 | 102.7 | 104.8 | 2.2 | #5 | 83.2 | 100.9 | 17.7 | #2 | 105.2 | 110.0 | 4.9 |
| #2 | 94.2 | 98.9 | 4.7 | #5 | 90.2 | 115.5 | 25.4 | #2 | 102.3 | 108.9 | 6.6 |
| #2 | 76.0 | 76.6 | 0.6 | #5 | 95.1 | 106.8 | 11.8 | #2 | 76.9 | 84.4 | 7.5 |
| #2 | 44.2 | 44.1 | -0.1 | #5 | 123.8 | 125.1 | 1.3 | #2 | 38.3 | 63.2 | 24.8 |
| #2 | 63.1 | 64.6 | 1.5 | #5 | 83.1 | 98.3 | 15.2 | #2 | 65.2 | 82.3 | 17.1 |
| #2 | 69.6 | 74.2 | 4.6 | #5 | 80.8 | 90.4 | 9.6 | #2 | 87.8 | 89.1 | 1.3 |
| #2 | 96.0 | 103.6 | 7.6 | #5 | 84.3 | 89.4 | 5.0 | #2 | 103.8 | 114.6 | 10.7 |
| #2 | 59.4 | 64.3 | 4.9 | #5 | 109.4 | 118.6 | 9.2 | #2 | 59.1 | 67.4 | 8.3 |
| #2 | 59.1 | 60.6 | 1.6 | #5 | 114.8 | 120.8 | 6.0 | #2 | 58.9 | 71.9 | 13.0 |
| #2 | 93.0 | 104.9 | 11.9 | #5 | 69.8 | 74.2 | 4.4 | #2 | 98.9 | 109.3 | 10.4 |
| #2 | 126.7 | 136.5 | 9.7 | #1 | 101.2 | 106.5 | 5.2 | #2 | 140.1 | 154.0 | 13.9 |
| #2 | 97.4 | 115.1 | 17.8 | #1 | 77.5 | 86.5 | 9.0 | #2 | 105.6 | 114.0 | 8.4 |
| #2 | 96.7 | 100.0 | 3.3 | #1 | 146.6 | 158.5 | 11.8 | #2 | 100.1 | 111.8 | 11.6 |
| #2 | 86.3 | 90.4 | 4.0 | #1 | 52.9 | 64.4 | 11.5 | #2 | 94.1 | 94.2 | 0.1 |
| #3 | 92.9 | 111.4 | 18.5 | #1 | 107.2 | 112.3 | 5.1 | #6 | 42.2 | 43.9 | 1.6 |
| #3 | 73.5 | 70.7 | -2.8 | #1 | 65.0 | 79.2 | 14.2 | #6 | 52.0 | 55.0 | 3.0 |
| #3 | 90.6 | 99.3 | 8.7 | #1 | 129.2 | 138.4 | 9.2 |  |  |  |  |
| #3 | 65.9 | 78.9 | 13.0 | #1 | 67.6 | 73.7 | 6.1 |  |  |  |  |
| #3 | 88.4 | 95.4 | 7.0 | #2 | 104.0 | 111.9 | 7.8 |  |  |  |  |
| #3 | 39.6 | 41.3 | 1.7 | #2 | 96.6 | 99.6 | 3.1 |  |  |  |  |
|  |  |  |  | #2 | 70.7 | 73.2 | 2.4 |  |  |  |  |
|  |  |  |  | #2 | 43.8 | 48.9 | 5.1 |  |  |  |  |
|  |  |  |  | #2 | 65.1 | 72.1 | 6.9 |  |  |  |  |
|  |  |  |  | #2 | 76.0 | 83.3 | 7.3 |  |  |  |  |
|  |  |  |  | #2 | 95.5 | 102.3 | 6.8 |  |  |  |  |
|  |  |  |  | #2 | 61.0 | 61.1 | 0.2 |  |  |  |  |
|  |  |  |  | #2 | 60.6 | 62.6 | 2.0 |  |  |  |  |
|  |  |  |  | #2 | 97.2 | 105.0 | 7.8 |  |  |  |  |
|  |  |  |  | #2 | 132.6 | 144.0 | 11.4 |  |  |  |  |
|  |  |  |  | #2 | 97.6 | 111.2 | 13.6 |  |  |  |  |
|  |  |  |  | #2 | 97.8 | 103.6 | 5.8 |  |  |  |  |
|  |  |  |  | #2 | 87.9 | 94.6 | 6.7 |  |  |  |  |
|  |  |  |  | #6 | 33.3 | 48.9 | 15.6 |  |  |  |  |
|  |  |  |  | #6 | 48.9 | 51.8 | 2.9 |  |  |  |  |
|  |  |  |  | #3 | 107.6 | 123.1 | 15.5 |  |  |  |  |
|  |  |  |  | #3 | 75.8 | 87.8 | 12.0 |  |  |  |  |
|  |  |  |  | #3 | 101.2 | 103.3 | 2.1 |  |  |  |  |
|  |  |  |  | #3 | 61.5 | 84.7 | 23.1 |  |  |  |  |
|  |  |  |  | #3 | 91.9 | 98.2 | 6.3 |  |  |  |  |
|  |  |  |  | #3 | 55.2 | 77.7 | 22.5 |  |  |  |  |
| **Count** | 34 | 34 | 34 |  | 54 | 54 | 54 |  | 30 | 30 | 30 |
| **Average** | 82.54 | 88.45 | 5.91 |  | 86.13 | 95.28 | 9.15 |  | 87.87 | 94.84 | 6.98 |
| **S.D.** | 20.18 | 23.29 | 5.18 |  | 24.48 | 24.80 | 6.02 |  | 24.66 | 24.55 | 5.58 |
| **S.E.M.** | 3.46 | 3.99 | 0.89 |  | 3.33 | 3.37 | 0.82 |  | 4.50 | 4.48 | 1.02 |

**Table S2.** **Raw data of surface area of individual alveoli (**$\boldsymbol{S}_{\boldsymbol{A}}$**) in Figure 3a.** Surface area of individual alveoli $\left( {\mu m}^{2} \right)$ in live lungs (3 mice for PEEP = 0 cmH_2_O; 6 mice for PEEP = 3 cmH_2_O; 3 mice for PEEP = 10 cmH_2_O).

| **PEEP = 0 [cmH_2_O]** | | | | **PEEP = 3 [cmH_2_O]** | | | | **PEEP = 10 [cmH_2_O]** | | | |
| --- | --- | --- | --- | --- | --- | --- | --- | --- | --- | --- | --- |
| Mouse No. | $S_{A\_Exp}$ [${\mu m}^{2}$] | $S_{A\_Insp}$ [${\mu m}^{2}$] | ${\Delta S}_{A}$ [${\mu m}^{2}$] | Mouse No. | $S_{A\_Exp}$ [${\mu m}^{2}$] | $S_{A\_Insp}$ [${\mu m}^{2}$] | ${\Delta S}_{A}$ [${\mu m}^{2}$] | Mouse No. | $S_{A\_Exp}$ [${\mu m}^{2}$] | $S_{A\_Insp}$ [${\mu m}^{2}$] | ${\Delta S}_{A}$ [${\mu m}^{2}$] |
| #1 | 34831 | 38631 | 3800.0 | #4 | 36150 | 39841 | 3691.2 | #1 | 39760 | 44290 | 4530.3 |
| #1 | 45990 | 56483 | 10493.0 | #4 | 25400 | 29056 | 3656.2 | #1 | 62028 | 67328 | 5300.0 |
| #1 | 39502 | 46765.2 | 7263.4 | #4 | 39265 | 44586 | 5320.8 | #1 | 43142 | 45583 | 2441.4 |
| #1 | 19988.3 | 22415.1 | 2426.8 | #4 | 36377 | 44842 | 8465.4 | #1 | 22917 | 22840.4 | -76.8 |
| #1 | 28574.9 | 34959.7 | 6384.8 | #4 | 15061 | 16686 | 1624.8 | #1 | 35404 | 38611.7 | 3207.8 |
| #1 | 17374.8 | 21223 | 3847.9 | #4 | 13142 | 17744 | 4601.9 | #1 | 23331 | 23043 | -288.7 |
| #1 | 13616 | 16069 | 2453.0 | #4 | 63357 | 68128 | 4771.0 | #1 | 15601 | 16849 | 1247.4 |
| #1 | 20708 | 21198 | 490.1 | #4 | 8452 | 10780 | 2328.1 | #1 | 24236 | 27791 | 3555.1 |
| #1 | 29272 | 30536 | 1264.2 | #5 | 16689 | 20031 | 3342.3 | #1 | 33704 | 35068 | 1364.4 |
| #1 | 14233 | 16297 | 2064.2 | #5 | 20274 | 26513 | 6239.3 | #1 | 22364 | 28640.9 | 6277.4 |
| #1 | 39130 | 47454 | 8324.3 | #5 | 36846 | 47751 | 10905.7 | #1 | 46955 | 55860 | 8905.0 |
| #1 | 15539 | 14578 | -961.0 | #5 | 35110 | 47898 | 12788.3 | #1 | 18337 | 20395 | 2058.4 |
| #1 | 42732 | 47427 | 4695.6 | #5 | 24783 | 30498 | 5715.3 | #1 | 45602.2 | 46912 | 1309.3 |
| #1 | 18024 | 20413 | 2388.7 | #5 | 21388 | 36372 | 14984.1 | #1 | 25829.1 | 29575.6 | 3746.5 |
| #2 | 38998.6 | 40754 | 1755.6 | #5 | 24488 | 36352 | 11863.8 | #2 | 42188 | 45550 | 3362.0 |
| #2 | 31752.6 | 34837 | 3084.1 | #5 | 32285 | 49155 | 16869.8 | #2 | 37059 | 41724 | 4664.9 |
| #2 | 25965.3 | 27080.9 | 1115.6 | #5 | 35596 | 44922 | 9326.9 | #2 | 26504 | 30192 | 3688.1 |
| #2 | 14958.2 | 15871.8 | 913.6 | #5 | 55420 | 58556 | 3136.0 | #2 | 16613 | 26041 | 9428.4 |
| #2 | 18261.1 | 22559.8 | 4298.7 | #5 | 24683 | 34575 | 9891.9 | #2 | 28809 | 32141 | 3331.2 |
| #2 | 34549 | 40124.6 | 5575.6 | #5 | 24287 | 30193 | 5906.2 | #2 | 39730 | 47390 | 7659.7 |
| #2 | 12877.1 | 15043.6 | 2166.5 | #5 | 24218 | 26862 | 2643.4 | #2 | 12705 | 16188 | 3482.6 |
| #2 | 15694.8 | 18058.2 | 2363.4 | #5 | 43217 | 52495 | 9278.1 | #2 | 15236 | 22803 | 7566.9 |
| #2 | 31712.6 | 40382.7 | 8670.1 | #5 | 46191 | 53747 | 7555.9 | #2 | 35719 | 43046 | 7327.0 |
| #2 | 60101 | 66609 | 6508.0 | #5 | 16315 | 19365 | 3049.9 | #2 | 72037 | 85022 | 12985.0 |
| #2 | 33711.9 | 46824 | 13111.9 | #1 | 35450 | 38944 | 3494.5 | #2 | 42755 | 47892 | 5137.8 |
| #2 | 32896.3 | 35738 | 2841.6 | #1 | 20941 | 26024 | 5082.4 | #2 | 36204 | 44744 | 8539.9 |
| #2 | 26591.9 | 29438 | 2846.3 | #1 | 73663 | 85968 | 12305.0 | #2 | 30979 | 30897 | -82.7 |
| #3 | 32933 | 43951 | 11018.0 | #1 | 10528 | 14621 | 4092.9 | #6 | 6983 | 7366 | 383.4 |
| #3 | 19961 | 20952 | 990.6 | #1 | 39635 | 43893 | 4258.1 | #6 | 9389 | 10709 | 1320.1 |
| #3 | 31520 | 36127 | 4607.1 | #1 | 15008 | 23024 | 8016.0 |  |  |  |  |
| #3 | 16858 | 23096 | 6238.5 | #1 | 59766 | 69206 | 9440.0 |  |  |  |  |
| #3 | 28837 | 33242 | 4405.1 | #1 | 17492 | 20735 | 3242.4 |  |  |  |  |
| #3 | 6235 | 7793 | 1558.1 | #2 | 41570 | 47367 | 5797.3 |  |  |  |  |
|  |  |  |  | #2 | 32952 | 34931 | 1978.9 |  |  |  |  |
|  |  |  |  | #2 | 62323 | 75511 | 13188.0 |  |  |  |  |
|  |  |  |  | #2 | 35164 | 43909 | 8745.1 |  |  |  |  |
|  |  |  |  | #2 | 33608 | 38758 | 5149.6 |  |  |  |  |
|  |  |  |  | #2 | 27511 | 31331 | 3820.6 |  |  |  |  |
|  |  |  |  | #6 | 4765 | 9122 | 4357.0 |  |  |  |  |
|  |  |  |  | #6 | 8477 | 9734 | 1256.9 |  |  |  |  |
|  |  |  |  | #3 | 43496 | 56200 | 12704.4 |  |  |  |  |
|  |  |  |  | #3 | 21389 | 29738 | 8349.8 |  |  |  |  |
|  |  |  |  | #3 | 38853 | 43012 | 4158.4 |  |  |  |  |
|  |  |  |  | #3 | 15224 | 27388 | 12164.4 |  |  |  |  |
|  |  |  |  | #3 | 30437 | 36618 | 6181.2 |  |  |  |  |
|  |  |  |  | #3 | 11223 | 21425 | 10201.4 |  |  |  |  |
| **Count** | 33 | 33 | 33 |  | 46 | 46 | 46 |  | 29 | 29 | 29 |
| **Average** | 27089 | 31301 | 4212 |  | 30401 | 37270 | 6868 |  | 31452 | 35672 | 4220 |
| **S.D.** | 11632 | 13760 | 3297 |  | 15731 | 17335 | 3915 |  | 15015 | 16796 | 3282 |
| **S.E.M.** | 2025 | 2395 | 574 |  | 2319 | 2556 | 577 |  | 2788 | 3119 | 609 |

**Table S3.** **Raw data of wall thickness of individual alveoli (**$\boldsymbol{T}_{\boldsymbol{A}}$**) in Figure 3b.** Wall thickness of individual alveoli $\left( \mu m \right)$ in live lungs (3 mice for PEEP = 0 cmH_2_O; 6 mice for PEEP = 3 cmH_2_O; 3 mice for PEEP = 10 cmH_2_O).

| **PEEP = 0 [cmH_2_O]** | | | | **PEEP = 3 [cmH_2_O]** | | | | **PEEP = 10 [cmH_2_O]** | | | |
| --- | --- | --- | --- | --- | --- | --- | --- | --- | --- | --- | --- |
| Mouse No. | $T_{A\_Exp}$ [$\mu m$] | $T_{A\_Insp}$ [$\mu m$] | ${\Delta T}_{A}$ [$\mu m$] | Mouse No. | $T_{A\_Exp}$ [$\mu m$] | $T_{A\_Insp}$ [$\mu m$] | ${\Delta T}_{A}$ [$\mu m$] | Mouse No. | $T_{A\_Exp}$ [$\mu m$] | $T_{A\_Insp}$ [$\mu m$] | ${\Delta T}_{A}$ [$\mu m$] |
| #1 | 17.79 | 13.80 | -4.0 | #4 | 13.96 | 12.49 | -1.5 | #1 | 11.87 | 7.54 | -4.3 |
| #1 | 13.30 | 12.11 | -1.2 | #4 | 12.27 | 11.58 | -0.7 | #1 | 8.47 | 6.65 | -1.8 |
| #1 | 12.45 | 10.87 | -1.6 | #4 | 23.10 | 21.17 | -1.9 | #1 | 9.24 | 8.83 | -0.4 |
| #1 | 11.76 | 11.96 | 0.2 | #4 | 19.38 | 10.93 | -8.5 | #1 | 10.24 | 10.91 | 0.7 |
| #1 | 16.99 | 13.27 | -3.7 | #4 | 18.01 | 11.66 | -6.4 | #1 | 8.39 | 10.24 | 1.9 |
| #1 | 19.00 | 12.09 | -6.9 | #4 | 16.11 | 10.31 | -5.8 | #1 | 11.56 | 14.93 | 3.4 |
| #1 | 22.14 | 18.93 | -3.2 | #4 | 21.41 | 15.26 | -6.2 | #1 | 18.55 | 14.14 | -4.4 |
| #1 | 21.21 | 21.73 | 0.5 | #4 | 26.76 | 12.16 | -14.6 | #1 | 10.86 | 17.43 | 6.6 |
| #1 | 13.38 | 7.46 | -5.9 | #5 | 12.80 | 9.39 | -3.4 | #1 | 9.16 | 9.84 | 0.7 |
| #1 | 23.66 | 17.30 | -6.4 | #5 | 13.49 | 7.58 | -5.9 | #1 | 17.57 | 19.15 | 1.6 |
| #1 | 13.82 | 21.51 | 7.7 | #5 | 12.60 | 7.96 | -4.6 | #1 | 10.23 | 8.33 | -1.9 |
| #1 | 24.39 | 18.72 | -5.7 | #5 | 7.55 | 5.96 | -1.6 | #1 | 13.76 | 18.15 | 4.4 |
| #1 | 12.86 | 11.82 | -1.0 | #5 | 10.39 | 8.19 | -2.2 | #1 | 11.03 | 10.60 | -0.4 |
| #1 | 26.57 | 30.85 | 4.3 | #5 | 12.85 | 8.69 | -4.2 | #1 | 23.47 | 20.35 | -3.1 |
| #2 | 11.43 | 8.79 | -2.6 | #5 | 12.93 | 11.39 | -1.5 | #2 | 7.40 | 5.96 | -1.4 |
| #2 | 17.06 | 19.62 | 2.6 | #5 | 21.97 | 15.13 | -6.8 | #2 | 8.14 | 12.86 | 4.7 |
| #2 | 24.52 | 21.97 | -2.6 | #5 | 15.37 | 16.18 | 0.8 | #2 | 17.52 | 24.39 | 6.9 |
| #2 | 16.09 | 19.09 | 3.0 | #5 | 8.16 | 8.02 | -0.1 | #2 | 15.51 | 16.14 | 0.6 |
| #2 | 19.74 | 17.94 | -1.8 | #5 | 12.01 | 10.79 | -1.2 | #2 | 21.46 | 14.03 | -7.4 |
| #2 | 23.43 | 23.54 | 0.1 | #5 | 9.06 | 9.21 | 0.2 | #2 | 13.83 | 10.97 | -2.9 |
| #2 | 14.45 | 15.21 | 0.8 | #5 | 18.95 | 16.57 | -2.4 | #2 | 14.44 | 13.83 | -0.6 |
| #2 | 16.08 | 14.30 | -1.8 | #5 | 19.83 | 16.72 | -3.1 | #2 | 18.16 | 10.40 | -7.8 |
| #2 | 21.02 | 22.16 | 1.1 | #5 | 14.05 | 11.30 | -2.8 | #2 | 20.71 | 19.61 | -1.1 |
| #2 | 13.70 | 19.75 | 6.1 | #5 | 13.37 | 10.98 | -2.4 | #2 | 19.61 | 15.97 | -3.6 |
| #2 | 19.53 | 18.20 | -1.3 | #1 | 13.61 | 10.21 | -3.4 | #2 | 19.22 | 10.20 | -9.0 |
| #2 | 17.00 | 22.66 | 5.7 | #1 | 14.53 | 11.71 | -2.8 | #2 | 18.92 | 16.90 | -2.0 |
| #2 | 16.14 | 16.38 | 0.2 | #1 | 12.40 | 9.74 | -2.7 | #2 | 14.44 | 16.93 | 2.5 |
| #2 | 16.96 | 16.62 | -0.3 | #1 | 10.52 | 9.72 | -0.8 | #2 | 12.61 | 18.84 | 6.2 |
| #3 | 18.96 | 14.44 | -4.5 | #1 | 15.36 | 11.85 | -3.5 | #6 | 12.17 | 10.11 | -2.1 |
| #3 | 15.62 | 15.50 | -0.1 | #1 | 17.33 | 14.17 | -3.2 | #6 | 7.59 | 9.86 | 2.3 |
| #3 | 10.88 | 12.55 | 1.7 | #1 | 17.20 | 23.25 | 6.1 |  |  |  |  |
| #3 | 8.94 | 17.02 | 8.1 | #1 | 22.93 | 18.70 | -4.2 |  |  |  |  |
| #3 | 10.52 | 7.24 | -3.3 | #1 | 11.15 | 9.88 | -1.3 |  |  |  |  |
| #3 | 9.89 | 13.56 | 3.7 | #1 | 19.91 | 16.37 | -3.5 |  |  |  |  |
|  |  |  |  | #1 | 13.65 | 12.53 | -1.1 |  |  |  |  |
|  |  |  |  | #1 | 19.21 | 16.23 | -3.0 |  |  |  |  |
|  |  |  |  | #1 | 13.45 | 13.88 | 0.4 |  |  |  |  |
|  |  |  |  | #1 | 22.43 | 22.87 | 0.4 |  |  |  |  |
|  |  |  |  | #2 | 9.38 | 7.06 | -2.3 |  |  |  |  |
|  |  |  |  | #2 | 22.24 | 18.41 | -3.8 |  |  |  |  |
|  |  |  |  | #2 | 24.46 | 22.00 | -2.5 |  |  |  |  |
|  |  |  |  | #2 | 15.59 | 15.79 | 0.2 |  |  |  |  |
|  |  |  |  | #2 | 19.90 | 18.70 | -1.2 |  |  |  |  |
|  |  |  |  | #2 | 23.82 | 17.88 | -5.9 |  |  |  |  |
|  |  |  |  | #2 | 16.83 | 15.53 | -1.3 |  |  |  |  |
|  |  |  |  | #2 | 16.31 | 14.37 | -1.9 |  |  |  |  |
|  |  |  |  | #2 | 24.47 | 26.07 | 1.6 |  |  |  |  |
|  |  |  |  | #2 | 19.41 | 15.41 | -4.0 |  |  |  |  |
|  |  |  |  | #2 | 19.92 | 15.91 | -4.0 |  |  |  |  |
|  |  |  |  | #2 | 22.18 | 23.04 | 0.9 |  |  |  |  |
|  |  |  |  | #2 | 18.13 | 13.46 | -4.7 |  |  |  |  |
|  |  |  |  | #2 | 13.22 | 14.48 | 1.3 |  |  |  |  |
|  |  |  |  | #6 | 7.33 | 4.22 | -3.1 |  |  |  |  |
|  |  |  |  | #6 | 7.88 | 5.02 | -2.9 |  |  |  |  |
|  |  |  |  | #3 | 16.27 | 12.39 | -3.9 |  |  |  |  |
|  |  |  |  | #3 | 14.42 | 11.96 | -2.5 |  |  |  |  |
|  |  |  |  | #3 | 14.64 | 12.09 | -2.6 |  |  |  |  |
|  |  |  |  | #3 | 15.83 | 10.92 | -4.9 |  |  |  |  |
|  |  |  |  | #3 | 14.43 | 14.97 | 0.5 |  |  |  |  |
|  |  |  |  | #3 | 10.54 | 10.38 | -0.2 |  |  |  |  |
| **Count** | 34 | 34 | 34 |  | 60 | 60 | 60 |  | 30 | 30 | 30 |
| **Average** | 16.80 | 16.44 | -0.36 |  | 15.95 | 13.35 | -2.61 |  | 13.87 | 13.47 | -0.40 |
| **S.D.** | 4.67 | 5.06 | 3.87 |  | 4.76 | 4.69 | 2.89 |  | 4.66 | 4.62 | 4.07 |
| **S.E.M.** | 0.80 | 0.87 | 0.66 |  | 0.61 | 0.61 | 0.37 |  | 0.85 | 0.84 | 0.74 |

**Table S4.** **Raw data of strain of alveoli (**$\boldsymbol{\varepsilon}_{\boldsymbol{A}}$**) shown in Figure 3c and of strain of entrance ring (**$\boldsymbol{\varepsilon}_{\boldsymbol{ER}}$**) shown in Figure 3d.** Strains measured in live lungs (3 mice for PEEP = 0 cmH_2_O; 6 mice for PEEP = 3 cmH_2_O; 3 mice for PEEP = 10 cmH_2_O).

| **PEEP = 0 [cmH_2_O]** | | | **PEEP = 3 [cmH_2_O]** | | | **PEEP = 10 [cmH_2_O]** | | |  |
| --- | --- | --- | --- | --- | --- | --- | --- | --- | --- |
| Mouse No. | $\varepsilon_{A}$ | $\varepsilon_{ER}$ | Mouse No. | $\varepsilon_{A}$ | $\varepsilon_{ER}$ | Mouse No. | $\varepsilon_{A}$ | $\varepsilon_{ER}$ | |
| #1 | 0.055 | 0.062 | #4 | 0.051 | 0.041 | #1 | 0.057 | 0.126 | |
| #1 | 0.114 | 0.069 | #4 | 0.072 | 0.041 | #1 | 0.043 | 0.078 | |
| #1 | 0.092 | 0.117 | #4 | 0.068 | 0.052 | #1 | 0.028 | 0.068 | |
| #1 | 0.061 | 0.144 | #4 | 0.116 | 0.016 | #1 | -0.002 | -0.018 | |
| #1 | 0.112 | 0.253 | #4 | 0.054 | 0.041 | #1 | 0.045 | -0.028 | |
| #1 | 0.111 | 0.251 | #4 | 0.175 | 0.075 | #1 | -0.006 | -0.061 | |
| #1 | 0.090 | 0.168 | #4 | 0.038 | 0.046 | #1 | 0.040 | -0.042 | |
| #1 | 0.012 | 0.147 | #4 | 0.138 | 0.068 | #1 | 0.073 | 0.043 | |
| #1 | 0.022 | 0.121 | #5 | 0.100 | 0.039 | #1 | 0.020 | 0.084 | |
| #1 | 0.073 | 0.219 | #5 | 0.154 | 0.053 | #1 | 0.140 | 0.188 | |
| #1 | 0.106 | 0.009 | #5 | 0.148 | 0.041 | #1 | 0.095 | 0.014 | |
| #1 | -0.031 | 0.092 | #5 | 0.182 | 0.063 | #1 | 0.056 | 0.001 | |
| #1 | 0.055 | 0.251 | #5 | 0.115 | 0.072 | #1 | 0.014 | 0.013 | |
| #1 | 0.066 | 0.136 | #5 | 0.350 | 0.098 | #1 | 0.073 | 0.049 | |
| #2 | 0.023 | -0.007 | #5 | 0.242 | 0.074 | #2 | 0.040 | 0.072 | |
| #2 | 0.049 | 0.109 | #5 | 0.261 | 0.057 | #2 | 0.063 | 0.003 | |
| #2 | 0.021 | 0.013 | #5 | 0.131 | 0.056 | #2 | 0.070 | 0.054 | |
| #2 | 0.031 | 0.483 | #5 | 0.028 | 0.020 | #2 | 0.284 | 0.256 | |
| #2 | 0.118 | 0.173 | #5 | 0.200 | 0.121 | #2 | 0.058 | 0.164 | |
| #2 | 0.081 | 0.072 | #5 | 0.122 | 0.077 | #2 | 0.096 | 0.052 | |
| #2 | 0.084 | 0.229 | #5 | 0.055 | 0.060 | #2 | 0.137 | 0.142 | |
| #2 | 0.075 | 0.124 | #5 | 0.107 | 0.046 | #2 | 0.248 | 0.195 | |
| #2 | 0.137 | 0.095 | #5 | 0.082 | 0.079 | #2 | 0.103 | 0.122 | |
| #2 | 0.054 | 0.109 | #5 | 0.093 | 0.043 | #2 | 0.090 | 0.034 | |
| #2 | 0.194 | 0.126 | #1 | 0.049 | 0.040 | #2 | 0.060 | 0.018 | |
| #2 | 0.043 | -0.023 | #1 | 0.121 | 0.057 | #2 | 0.118 | 0.234 | |
| #2 | 0.054 | 0.090 | #1 | 0.084 | 0.097 | #2 | -0.001 | 0.195 | |
| #3 | 0.167 | 0.353 | #1 | 0.194 | 0.060 | #6 | 0.027 | 0.031 | |
| #3 | 0.025 | 0.259 | #1 | 0.054 | 0.059 | #6 | 0.070 | 0.037 | |
| #3 | 0.073 | 0.226 | #1 | 0.267 | 0.079 |  |  |  | |
| #3 | 0.185 | 0.297 | #1 | 0.079 | 0.061 |  |  |  | |
| #3 | 0.076 | -0.056 | #1 | 0.093 | 0.056 |  |  |  | |
| #3 | 0.125 | 0.235 | #2 | 0.070 | 0.064 |  |  |  | |
|  |  |  | #2 | 0.030 | 0.042 |  |  |  | |
|  |  |  | #2 | 0.106 | 0.040 |  |  |  | |
|  |  |  | #2 | 0.124 | 0.063 |  |  |  | |
|  |  |  | #2 | 0.077 | 0.049 |  |  |  | |
|  |  |  | #2 | 0.069 | 0.048 |  |  |  | |
|  |  |  | #6 | 0.457 | 0.060 |  |  |  | |
|  |  |  | #6 | 0.074 | 0.070 |  |  |  | |
|  |  |  | #3 | 0.146 | 0.080 |  |  |  | |
|  |  |  | #3 | 0.195 | 0.052 |  |  |  | |
|  |  |  | #3 | 0.054 | 0.074 |  |  |  | |
|  |  |  | #3 | 0.400 | 0.067 |  |  |  | |
|  |  |  | #3 | 0.102 | 0.063 |  |  |  | |
|  |  |  | #3 | 0.454 | 0.075 |  |  |  | |
| **Count** | 33 | 33 |  | 46 | 46 |  | 29 | 29 | |
| **Average** | 0.077 | 0.150 |  | 0.139 | 0.060 |  | 0.074 | 0.073 | |
| **S.D.** | 0.050 | 0.114 |  | 0.105 | 0.019 |  | 0.066 | 0.084 | |
| **S.E.M.** | 0.00873 | 0.01979 |  | 0.015 | 0.003 |  | 0.012 | 0.016 | |

**Table S5.** **Raw data of q shown in Figure 4i.**

| **PEEP = 0 [cmH_2_O]** | | | | | | | |
| --- | --- | --- | --- | --- | --- | --- | --- |
| Mouse No. | $S_{Exp}$ [${\mu m}^{2}$] | $V_{Exp}$ [${\mu m}^{3}$] | $k_{Exp}$ | $S_{Exp}$ [${\mu m}^{2}$] | $V_{Exp}$ [${\mu m}^{3}$] | $k_{Exp}$ | $q=k_{Insp}/k_{Exp}$ |
| #1 | 565961 | 14133947 | 9.6815 | 620202 | 16555230 | 9.5479 | 0.9862 |
| #1 | 347719 | 8909570 | 8.0908 | 382395 | 10682956 | 7.8835 | 0.9744 |
| #1 | 415614 | 9309054 | 9.3919 | 454443 | 11381861 | 8.9813 | 0.9563 |
| #1 | 783496 | 21746598 | 10.0564 | 844248 | 26167790 | 9.5784 | 0.9525 |
| #2 | 454944 | 11428682 | 8.9666 | 492590 | 13603323 | 8.6441 | 0.9640 |
| #2 | 568118 | 15933936 | 8.9720 | 628211 | 19839020 | 8.5722 | 0.9554 |
| #2 | 487731 | 13493859 | 8.6051 | 551318 | 16466026 | 8.5181 | 0.9899 |
| #3 | 376781 | 6676982 | 10.6260 | 440791 | 10997416 | 8.9133 | 0.8388 |
| #3 | 576482 | 10827721 | 11.7786 | 662804 | 16120073 | 10.3866 | 0.8818 |
| #3 | 681164 | 15001228 | 11.1987 | 802998 | 21167452 | 10.4939 | 0.9371 |
| **Count** |  |  |  |  |  |  | 10 |
| **Average** |  |  |  |  |  |  | 0.9436 |
| **S.D.** |  |  |  |  |  |  | 0.0478 |
| **S.E.M.** |  |  |  |  |  |  | 0.0151 |
| **PEEP = 3 [cmH_2_O]** | | | | | | | |
| Mouse No. | $S_{Exp}$ [${\mu m}^{2}$] | $V_{Exp}$ [${\mu m}^{3}$] | $k_{Exp}$ | $S_{Exp}$ [${\mu m}^{2}$] | $V_{Exp}$ [${\mu m}^{3}$] | $k_{Exp}$ | $q=k_{Insp}/k_{Exp}$ |
| #1 | 740810 | 21091522 | 9.7044 | 823290 | 24153068 | 9.8531 | 1.0153 |
| #1 | 502415 | 12579173 | 9.2888 | 569030 | 14782171 | 9.4473 | 1.0171 |
| #1 | 778869 | 22887382 | 9.6620 | 855789 | 25921272 | 9.7708 | 1.0113 |
| #1 | 722204 | 20654096 | 9.5938 | 791948 | 23310864 | 9.7049 | 1.0116 |
| #2 | 264289 | 6012375 | 7.9931 | 320605 | 8025888 | 7.9979 | 1.0006 |
| #2 | 338725 | 7868088 | 8.5625 | 434438 | 10486262 | 9.0680 | 1.0590 |
| #2 | 314352 | 6653333 | 8.8863 | 393592 | 8652290 | 9.3388 | 1.0509 |
| #2 | 318434 | 7049709 | 8.6611 | 416754 | 10128233 | 8.9027 | 1.0279 |
| #2 | 923857 | 26447188 | 10.4076 | 1011225 | 29153396 | 10.6755 | 1.0257 |
| #2 | 831636 | 22859540 | 10.3250 | 941571 | 26179018 | 10.6795 | 1.0343 |
| #2 | 522245 | 13881729 | 9.0416 | 591997 | 16394169 | 9.1733 | 1.0146 |
| #2 | 760067 | 19286428 | 10.5686 | 833907 | 21477378 | 10.7927 | 1.0212 |
| #3 | 581718 | 14927730 | 9.5951 | 656014 | 17610614 | 9.6916 | 1.0101 |
| #3 | 362050 | 9708608 | 7.9554 | 402001 | 11181555 | 8.0394 | 1.0106 |
| #3 | 407811 | 9834102 | 8.8846 | 459389 | 11554038 | 8.9886 | 1.0117 |
| #3 | 798917 | 23551028 | 9.7236 | 896632 | 27329918 | 9.8822 | 1.0163 |
| #4 | 451035 | 12544048 | 8.3545 | 513095 | 15223028 | 8.3534 | 0.9999 |
| #4 | 571977 | 18232050 | 8.2570 | 662279 | 21970492 | 8.4427 | 1.0225 |
| #4 | 491302 | 15154475 | 8.0227 | 567983 | 18026046 | 8.2617 | 1.0298 |
| #5 | 614010 | 13383933 | 10.8923 | 657403 | 14622984 | 10.9936 | 1.0093 |
| #6 | 488908 | 11166949 | 9.7860 | 597520 | 13754283 | 10.4086 | 1.0636 |
| #6 | 677584 | 14895193 | 11.1926 | 763618 | 17538854 | 11.3121 | 1.0107 |
| #6 | 824065 | 20212944 | 11.1056 | 987779 | 24569558 | 11.6877 | 1.0524 |
| **Count** |  |  |  |  |  |  | 23 |
| **Average** |  |  |  |  |  |  | 1.0229 |
| **S.D.** |  |  |  |  |  |  | 0.0180 |
| **S.E.M.** |  |  |  |  |  |  | 0.0037 |
| **PEEP = 10 [cmH_2_O]** | | | | | | | |
| Mouse No. | $S_{Exp}$ [${\mu m}^{2}$] | $V_{Exp}$ [${\mu m}^{3}$] | $k_{Exp}$ | $S_{Exp}$ [${\mu m}^{2}$] | $V_{Exp}$ [${\mu m}^{3}$] | $k_{Exp}$ | $q=k_{Insp}/k_{Exp}$ |
| #1 | 635201 | 16753912 | 9.7014 | 674138 | 18112324 | 9.7746 | 1.0075 |
| #1 | 381092 | 10310329 | 8.0448 | 405258 | 11186046 | 8.1024 | 1.0072 |
| #1 | 450114 | 10855741 | 9.1808 | 485592 | 12147114 | 9.1894 | 1.0009 |
| #1 | 860906 | 25674992 | 9.8920 | 905394 | 27205964 | 10.0091 | 1.0118 |
| #2 | 509659 | 15026218 | 8.3698 | 568843 | 17873864 | 8.3211 | 0.9942 |
| #2 | 656109 | 21573448 | 8.4664 | 731968 | 25274744 | 8.4990 | 1.0039 |
| #2 | 560030 | 17425158 | 8.3322 | 631223 | 20534616 | 8.4177 | 1.0103 |
| #6 | 634420 | 14066389 | 10.8873 | 684514 | 16665990 | 10.4913 | 0.9636 |
| **Count** |  |  |  |  |  |  | 8 |
| **Average** |  |  |  |  |  |  | 0.9999 |
| **S.D.** |  |  |  |  |  |  | 0.0157 |
| **S.E.M.** |  |  |  |  |  |  | 0.0056 |

**Table S6.** **The angle *theta (***$\boldsymbol{\theta}$***)* between two representative neighboring alveoli** The angle, theta, between representative two neighboring alveoli. Measured data were 22 sets of alveoli over 3 live mice at PEEP = 0 cmH_2_O, 92 sets of alveoli over 6 live mice at PEEP = 3 cmH_2_O, and 22 sets of alveoli over 3 live mice at PEEP = 10 cmH_2_O. The average $\Delta\theta$ were 27.4 $\pm$3.5^o^ (mean$\pm$s.e.m), -17.0 $\pm$1.7^o^ (mean$\pm$s.e.m), and 2.0 $\pm$3.05^o^ (mean$\pm$s.e.m), respectively.

| **PEEP = 0 [cmH_2_O]** | | | |
| --- | --- | --- | --- |
| Mouse No | $\theta_{Exp}$ | $\theta_{Insp}$ | $\Delta\theta(=\theta_{Insp}-\theta_{Exp})$ [$^{\circ}$] |
| #1 | 73.2 | 117.4 | 44.2 |
| #1 | 142 | 164.9 | 22.9 |
| #1 | 91.2 | 101 | 9.8 |
| #1 | 59.8 | 114.9 | 55.1 |
| #2 | 46.4 | 90.3 | 43.9 |
| #2 | 69.3 | 81.3 | 12 |
| #2 | 91.8 | 109.8 | 18 |
| #2 | 70.1 | 104.3 | 34.2 |
| #2 | 60.2 | 82.2 | 22 |
| #2 | 55.6 | 87.9 | 32.3 |
| #2 | 126.3 | 131.2 | 4.9 |
| #2 | 64.3 | 82.8 | 18.5 |
| #2 | 113 | 139.5 | 26.5 |
| #3 | 48.3 | 108.5 | 60.2 |
| #3 | 112.7 | 141.8 | 29.1 |
| #3 | 53.7 | 67.9 | 14.2 |
| #3 | 27.3 | 65.9 | 38.6 |
| #3 | 47.8 | 64.4 | 16.6 |
| #3 | 42.7 | 35.5 | -7.2 |
| #3 | 87.2 | 132.4 | 45.2 |
| #3 | 53.3 | 95.6 | 42.3 |
| #3 | 20.1 | 40.2 | 20.1 |
| **Count** | 22 | 22 | 22 |
| **Average** | 70.7 | 98.2 | 27.4 |
| **S.D.** | 30.8 | 32.1 | 16.4 |
| **S.E.M.** | 6.6 | 6.8 | 3.5 |
| **PEEP = 3 [cmH_2_O]** | | | |
| Mouse No | $\theta_{Exp}$ | $\theta_{Insp}$ | $\Delta\theta(=\theta_{Insp}-\theta_{Exp})$ [$^{\circ}$] |
| #4 | 102.5 | 88.8 | -13.7 |
| #4 | 87.8 | 65.2 | -22.6 |
| #4 | 115.4 | 88.5 | -26.9 |
| #4 | 30.8 | 14.8 | -16 |
| #4 | 125.5 | 100.3 | -25.2 |
| #4 | 127.2 | 92.3 | -34.9 |
| #4 | 71 | 67 | -4 |
| #4 | 142.8 | 131.6 | -11.2 |
| #4 | 101.3 | 110 | 8.7 |
| #4 | 100.4 | 50.4 | -50 |
| #4 | 101.1 | 104.5 | 3.4 |
| #4 | 78.3 | 58.1 | -20.2 |
| #4 | 62.1 | 39.9 | -22.2 |
| #4 | 29.8 | 33.3 | 3.5 |
| #4 | 51 | 40.3 | -10.7 |
| #4 | 109.5 | 83.8 | -25.7 |
| #5 | 108.7 | 114.3 | 5.6 |
| #5 | 84 | 93.7 | 9.7 |
| #5 | 75.7 | 89.2 | 13.5 |
| #5 | 125 | 114 | -11 |
| #5 | 94.2 | 80.1 | -14.1 |
| #5 | 111.6 | 70.5 | -41.1 |
| #5 | 126 | 105.9 | -20.1 |
| #5 | 91.7 | 70.9 | -20.8 |
| #5 | 84.8 | 71.1 | -13.7 |
| #5 | 83.8 | 70.9 | -12.9 |
| #5 | 54.7 | 27.4 | -27.3 |
| #5 | 84.2 | 57.4 | -26.8 |
| #5 | 97.5 | 97.3 | -0.2 |
| #5 | 94.7 | 84.8 | -9.9 |
| #5 | 112.6 | 106.1 | -6.5 |
| #5 | 144.6 | 111.3 | -33.3 |
| #5 | 76.2 | 50.7 | -25.5 |
| #5 | 89 | 81.4 | -7.6 |
| #5 | 94.8 | 75.7 | -19.1 |
| #5 | 106.6 | 49 | -57.6 |
| #5 | 127.1 | 103.3 | -23.8 |
| #5 | 61.9 | 39.2 | -22.7 |
| #5 | 65.2 | 32.5 | -32.7 |
| #5 | 113.2 | 83.5 | -29.7 |
| #5 | 116.5 | 91.5 | -25 |
| #5 | 101.1 | 84.5 | -16.6 |
| #5 | 89.1 | 78.6 | -10.5 |
| #5 | 54.2 | 54.6 | 0.4 |
| #5 | 42.5 | 33.2 | -9.3 |
| #5 | 113 | 73.1 | -39.9 |
| #5 | 39.6 | 25.8 | -13.8 |
| #5 | 104.2 | 75.2 | -29 |
| #1 | 72.6 | 70.1 | -2.5 |
| #1 | 29 | 30 | 1 |
| #1 | 46 | 39.5 | -6.5 |
| #1 | 68.1 | 56.6 | -11.5 |
| #1 | 142.4 | 103 | -39.4 |
| #1 | 115.4 | 99.7 | -15.7 |
| #1 | 83.4 | 86.8 | 3.4 |
| #1 | 80.1 | 82.6 | 2.5 |
| #1 | 138 | 123.9 | -14.1 |
| #1 | 66.8 | 60.2 | -6.6 |
| #1 | 132.7 | 115.8 | -16.9 |
| #1 | 91.3 | 80.5 | -10.8 |
| #1 | 116.1 | 85.7 | -30.4 |
| #1 | 92.7 | 110.1 | 17.4 |
| #1 | 128.2 | 93.3 | -34.9 |
| #1 | 132.1 | 104.8 | -27.3 |
| #2 | 40.3 | 58.2 | 17.9 |
| #2 | 51 | 63.4 | 12.4 |
| #2 | 58.4 | 62.3 | 3.9 |
| #2 | 61.5 | 55.3 | -6.2 |
| #2 | 84.6 | 64.2 | -20.4 |
| #2 | 114.9 | 103 | -11.9 |
| #2 | 98 | 84.7 | -13.3 |
| #2 | 127.65 | 92.8 | -34.85 |
| #2 | 108.2 | 100.1 | -8.1 |
| #2 | 72.1 | 60.7 | -11.4 |
| #2 | 131.9 | 123.5 | -8.4 |
| #2 | 93.9 | 41.3 | -52.6 |
| #6 | 134.4 | 107.9 | -26.5 |
| #6 | 134.9 | 109.9 | -25 |
| #6 | 89.3 | 92.8 | 3.5 |
| #6 | 68.3 | 41.9 | -26.4 |
| #3 | 109 | 58.2 | -50.8 |
| #3 | 78.2 | 35.4 | -42.8 |
| #3 | 65.4 | 45.9 | -19.5 |
| #3 | 97.2 | 58.1 | -39.1 |
| #3 | 40.7 | 34.7 | -6 |
| #3 | 45.6 | 44.1 | -1.5 |
| #3 | 72.9 | 48.5 | -24.4 |
| #3 | 37.7 | 36.3 | -1.4 |
| #3 | 142.6 | 97.6 | -45 |
| #3 | 65.4 | 35.7 | -29.7 |
| #3 | 82.7 | 64.6 | -18.1 |
| #3 | 89.1 | 41.3 | -47.8 |
| **Count** | 92 | 92 | 92 |
| **Average** | 90.6 | 73.6 | -17.0 |
| **S.D.** | 30.1 | 27.6 | 16.4 |
| **S.E.M.** | 3.1 | 2.9 | 1.7 |
| **PEEP = 10 [cmH_2_O]** | | | |
| Mouse No | $\theta_{Exp}$ | $\theta_{Insp}$ | $\Delta\theta(=\theta_{Insp}-\theta_{Exp})$ [$^{\circ}$] |
| #1 | 63.6 | 59.1 | -4.5 |
| #1 | 91.6 | 85 | -6.6 |
| #1 | 40 | 48.6 | 8.6 |
| #1 | 47.7 | 54.1 | 6.4 |
| #2 | 110 | 118.3 | 8.3 |
| #2 | 69.2 | 69.6 | 0.4 |
| #2 | 95.7 | 101.7 | 6 |
| #2 | 50.9 | 53.3 | 2.4 |
| #2 | 112.8 | 123.7 | 10.9 |
| #2 | 40.2 | 32.1 | -8.1 |
| #2 | 63.7 | 63.8 | 0.1 |
| #2 | 134.9 | 143.7 | 8.8 |
| #2 | 33.8 | 39.2 | 5.4 |
| #6 | 105.3 | 102.7 | -2.6 |
| #6 | 42.9 | 45 | 2.1 |
| #6 | 127.1 | 124.2 | -2.9 |
| #6 | 81.4 | 63.5 | -17.9 |
| #6 | 49.5 | 35.1 | -14.4 |
| #6 | 107.5 | 135.4 | 27.9 |
| #6 | 130.5 | 104.3 | -26.2 |
| #6 | 81.3 | 76.9 | -4.4 |
| #6 | 36.4 | 81.2 | 44.8 |
| **Count** | 22 | 22 | 22 |
| **Average** | 78 | 80.0 | 2.0 |
| **S.D.** | 32.7 | 33.5 | 14.3 |
| **S.E.M.** | 7.0 | 7.1 | 3.1 |
